# Fascinating Furanosteroids and Their Pharmacological Profile

**DOI:** 10.3390/molecules28155669

**Published:** 2023-07-26

**Authors:** Valery M. Dembitsky

**Affiliations:** Centre for Applied Research, Innovation and Entrepreneurship, Lethbridge College, 3000 College Drive South, Lethbridge, AB T1K 1L6, Canada; valery.dembitsky@lethbridgecollege.ca

**Keywords:** furanosteroids, isoprenoid lipids, fungi, fungal endophytes, plants, marine organisms

## Abstract

This review article delves into the realm of furanosteroids and related isoprenoid lipids derived from diverse terrestrial and marine sources, exploring their wide array of biological activities and potential pharmacological applications. Fungi, fungal endophytes, plants, and various marine organisms, including sponges, corals, molluscs, and other invertebrates, have proven to be abundant reservoirs of these compounds. The biological activities exhibited by furanosteroids and related lipids encompass anticancer, cytotoxic effects against various cancer cell lines, antiviral, and antifungal effects. Notably, the discovery of exceptional compounds such as nakiterpiosin, malabaricol, dysideasterols, and cortistatins has revealed their potent anti-tuberculosis, antibacterial, and anti-hepatitis C attributes. These compounds also exhibit activity in inhibiting protein kinase C, phospholipase A2, and eliciting cytotoxicity against cancer cells. This comprehensive study emphasizes the significance of furanosteroids and related lipids as valuable natural products with promising therapeutic potential. The remarkable biodiversity found in both terrestrial and marine ecosystems offers an extensive resource for unearthing novel biologically active compounds, paving the way for future drug development and advancements in biomedical research. This review presents a compilation of data obtained from various studies conducted by different authors who employed the PASS software 9.1 to evaluate the biological activity of natural furanosteroids and compounds closely related to them. The utilization of the PASS software in this context offers valuable advantages, such as screening large chemical libraries, identifying compounds for subsequent experimental investigations, and gaining insights into potential biological activities based on their structural features. Nevertheless, it is crucial to emphasize that experimental validation remains indispensable for confirming the predicted activities.

## 1. Introduction

Natural and synthesized organic compounds that contain a furan ring(s) exhibit diverse cardiovascular activity [1,2,3]. These compounds find extensive use as antibacterial, antiviral, anti-inflammatory, antifungal, anticancer, antihyperglycemic, analgesic, and anticonvulsant medications [1,2,3,4,5,6]. According to *ChemNetBase*, the number of natural compounds containing a furan ring exceeds 12,000 per molecule. Additionally, compounds with a 2,5-dihydrofuran ring surpass 12,200, while those with a 2,3-dihydrofuran ring exceed 1850. Moreover, compounds with a tetrahydrofuran ring account for over 47,570 compounds. Furthermore, steroids and related isoprenoid lipids that possess the additional ring(s) can be referred to as furanosteroids and their analogues and derivatives, comprising a collection of over 1000 compounds [7].

Furanosteroids represent a class of pentacyclic isoprenoid lipids that are synthesized by fungi and various other organisms. These compounds possess additional fused furan, dihydrofuran, or tetrahydrofuran ring(s) attached to their pentacyclic backbone [8,9,10].

Initially, furanosteroids were identified in mushrooms nearly 80 years ago, characterized by the presence of an extra furan ring connecting positions 4 and 6 of the steroid skeleton. However, the term “*furanosteroids*” has since been expanded to include any steroids and related compounds that contain the furan ring, 2,3-dihydrofuran ring, 2,5-dihydrofuran ring, and/or tetrahydrofuran ring (s). It is believed that the strained furan cycle contributes to the diverse biological activities exhibited by these compounds. Several metabolites belonging to this class of natural products have garnered significant attention in pharmacological research due to their potent anti-inflammatory and antibiotic properties, potential anti-proliferative activity, and ability to inhibit inositide-3-kinase [8,9,11,12,13,14].

This review is dedicated to furanosteroids and related isoprenoid lipids, their occurrence in fungi, plants, and marine organisms, and the exploration of their biological activity.

## 2. Furanosteroids Produced by Fungi and Fungal Endophytes

**Figure 1:**
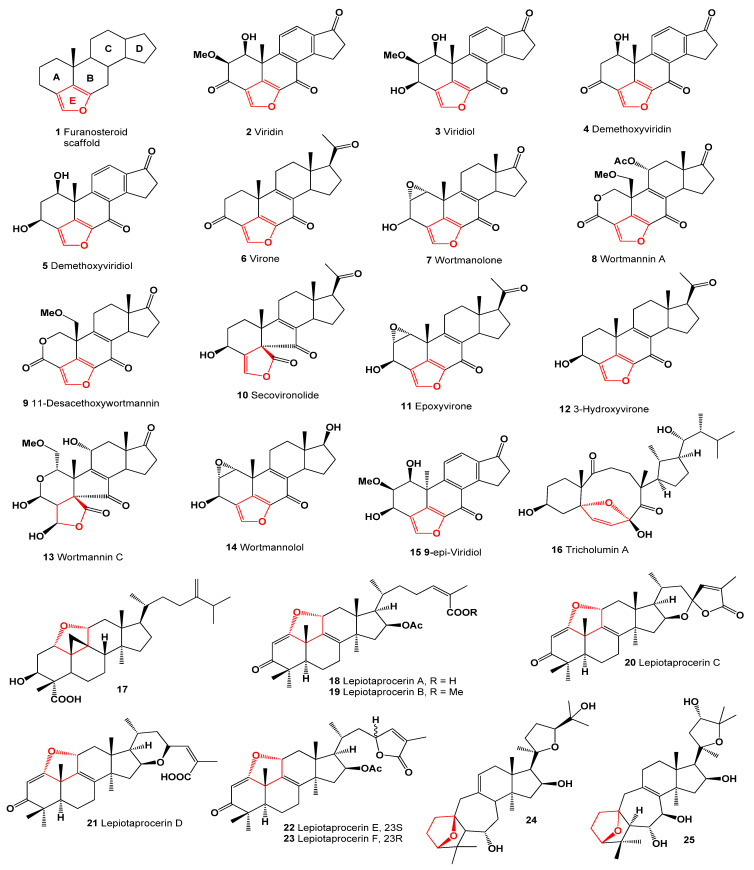
Furanosteroids produced by fungal species.

**Figure 2:**
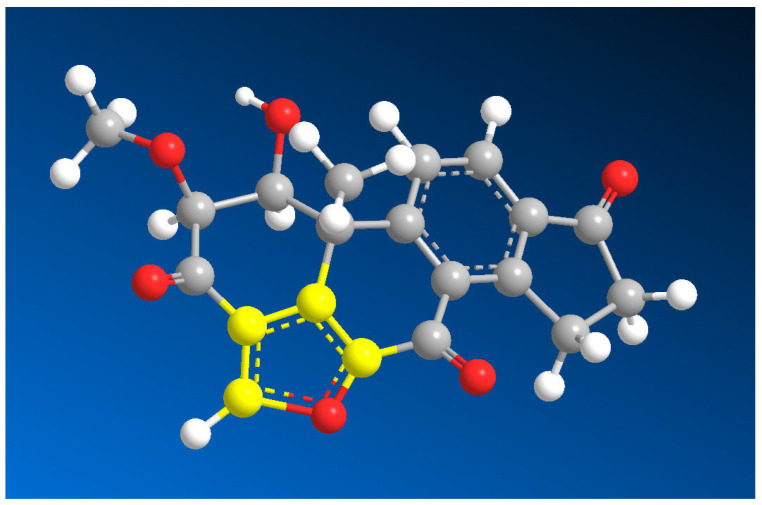
Three-dimensional structure of furanosteroid named viridin (**2**) and showing a wide range of antifungal and other biological activities. A distinctive characteristic of natural furanosteroids, as was believed in the 1950s of the last century, is the presence of an additional aromatic furan ring in the structure of the steroid. The aromatic furane ring is highlighted in yellow. The percentage of biological activities is shown in Figure 3. Viridin, an antibiotic produced by a pigment-forming strain of the common soil fungus *Trichoderma viride*, was first described by Brian and McGowan, and Brian, Curtis, Hemming and McGowan. Viridin is not antibacterial but is highly antifungal. Its activity against certain fungi is remarkably high. Germination of the spores of, for example, *Botrytis allii* is prevented by a concentration of 0.019 p.p.m. of α-viridin or 0.156 p.p.m. of β-viridin. Among synthetic fungicides, only the organo-mercurials are of the same order of activity. gray is carbon, white is hydrogen, and red is oxygen.

The furanosteroid scaffold (**1**) represents a highly oxygenated framework comprising a [5,6,6,6]-tetracycle (A-B-C-D rings), along with the presence of a furan ring, as depicted in Figure 1. Furanosteroids, exemplified by compounds **2**–**9**, **11**, **12**, **14**, and **15**, with their biological activities summarized in Table 1, encompass both the furan ring and steroids, viridin (**2**, 3D model is shown in Figure 2), **3**–**5**, and **15**.

These steroids can be classified as aromatic steroids due to the presence of the C aromatic ring. Among them, the steroid known as secovironolide (**10**) contains an additional 2,3-dihydrofuran ring, while tricholumin A (**16**) is an example that includes a 2,5-dihydrofuran ring. Furthermore, the most commonly encountered steroids, **13** and **17**–**25**, feature a tetrahydrofuran ring, as illustrated in Figure 1.

The data presented in Table 1, Table 2, Table 3, Table 4, Table 5, Table 6, Table 7, Table 8, Table 9 and Table 10 are taken from published data and obtained using the German computer software PASS (http://www.akosgmbh.de/mobile/pass.htm accessed on 1 April 2023). This program is in the public domain, and annually it is used by more than 26,000 scientists from around the world. The site of this program provides complete information on its use as well as the interpretation of the data obtained.

**Figure 3:**
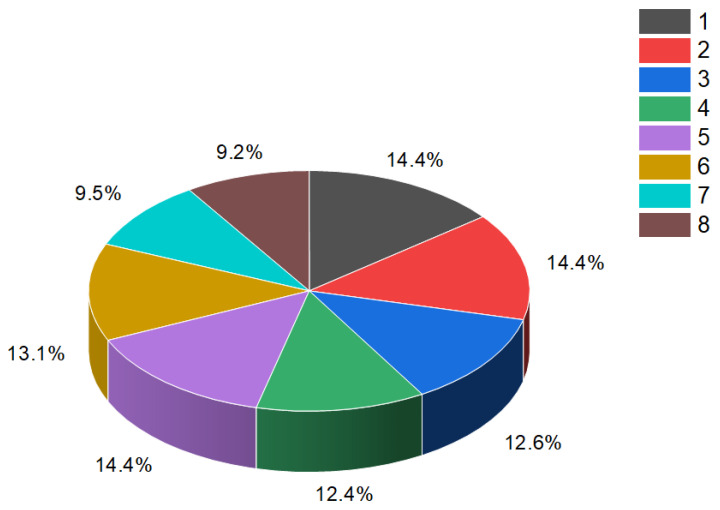
Percentage distribution of dominant and related biological activities using the widely recognized furanosteroid, viridin (**2**), which possesses unique pharmacological properties. The activities are indicated by the following numbers: 1. *Antifungal* (14.4%); 2. *Anti-inflammatory* (14.4%); 3. *Antiprotozoal (Plasmodium)* (12.6%); 4. *Antibacterial* (12.4%); 5. *Antineoplastic* (14.4%); 6. *Apoptosis agonist* (13.1%); 7. *Prostate disorders treatment* (9.5%); and 8. *Proliferative diseases treatment* (9.2%). The antifungal activity of the fungus *Trichoderma viride* has been observed to exhibit an inhibitory effect against various pathogens, such as *Fusarium solani, Rhizoctonia solani*, and *Sclerotium rolfsii*. Moreover, the extract obtained from raw mycelium has demonstrated notable antimicrobial activity, specifically displaying an antibacterial effect against *Bacillus subtilis, Escherichia coli*, and *Pseudomonas fluorescens*. Maximum antifungal effectiveness has also been recorded against *Candida albicans, Rhizoctonia solani, Pythium ultimum, Fusarium solani*, and *F. oxysporium*. The viridin metabolite present in the alcoholic mycelia extract of *Trichoderma viride* exerts antimicrobial, antifungal, and anticancer effects [14,32,33,34].

**Figure 4:**
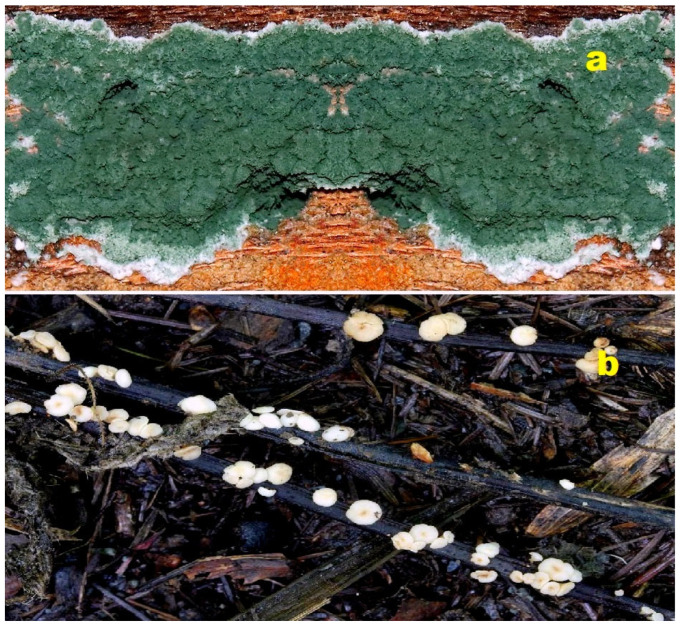
(**a**) *Trichoderma viride* is a fungus or mold that reproduces asexually through spores. The mycelium of this fungus can produce various enzymes, such as cellulases and chitinases. These enzymes are involved in the degradation of cellulose and chitin, respectively. *Trichoderma viride* commonly grows on wood, which predominantly consists of cellulose, as well as on fungi whose cell walls are primarily composed of chitin. It exhibits a parasitic nature towards the mycelium and fruiting bodies of other fungi, including cultivated ones. The fungus is known to cause “*Green mold fungus disease*” [38,39]. (**b**) *Hymenoscyphus pseudoalbidus*, also known as the Ash Wax Moth, is a saprotrophic fungus. It can be found growing on decaying trunks and branches of ash trees. The fruiting bodies of this fungus are hardly distinguishable from a macroscopic or microscopic perspective. It is commonly observed on the same substrate [40,41].

Viridin (**2**, activity is shown in Figure 3), a furano-steroidal antibiotic, represents the first identified member of the furanosteroid family. It was initially isolated in 1945 from a pigment-forming strain of the common soil fungus *Trichoderma viride* (as shown in Figure 4) [15,16]. Viridin, along with its derivatives **3**, **4**, **5**, **6**, and **11**, have demonstrated remarkable antifungal and antibacterial activities. Moreover, these compounds are known as potent inhibitors of the lipid kinase PI-3K [16]. Phosphatidylinositol 3-kinases (PI3Ks) are lipid kinases that play a central role in cell cycle regulation, apoptosis, DNA repair, aging, angiogenesis, cellular metabolism, and motility [16,17,18]. These enzymes catalyze the synthesis of specific members of the lipid family collectively known as “phosphoinositides”. These PI3K products can in turn modulate the activation of many downstream proteins, ultimately regulating several cellular processes. Mammalian cells possess eight PI3Ks, which are grouped into three classes depending on their structure and substrate specificity [16,17,18].

**Figure 5:**
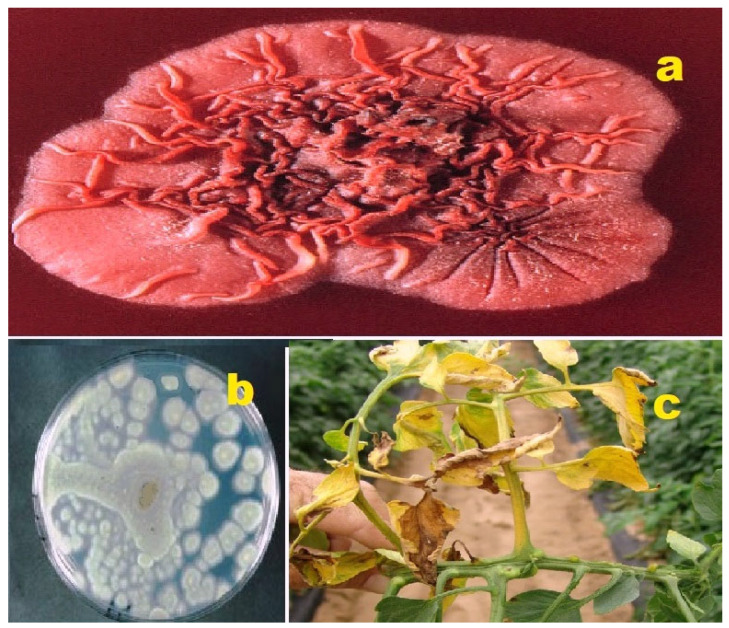
(**a**) *Talaromyces marneffei*: Talaromyces is a genus of fungi belonging to the family Trichocomaceae. It was first described in 1955 by the American mycologist Chester Ray Benjamin, who specialized in the taxonomy of fungal molds in the Eurotiales and Mucorales orders. *Talaromyces marneffei*, previously known as *Penicillium marneffei*, was identified in 1956. This fungus is endemic to Southeast Asia and is a significant cause of opportunistic infections in individuals with immunodeficiency associated with HIV/AIDS [42,43]. (**b**) *Penicillium funiculosum*: *P. funiculosum* is a plant pathogenic fungus that infects pineapple fruits. The disease it causes is referred to as fruit core rot, which is characterized by the browning or blackening and rotting of the fruit’s core [44,45]. (**c**) *Fusarium oxysporum*: *F. oxysporum* is an ascomycete fungus belonging to the family Nectriaceae. *F. oxysporum* strains are commonly found in soil and can exist as saprophytes, deriving nutrients from decaying organic matter. They are capable of degrading lignin and complex carbohydrates present in soil residues [46,47].

**Figure 6:**
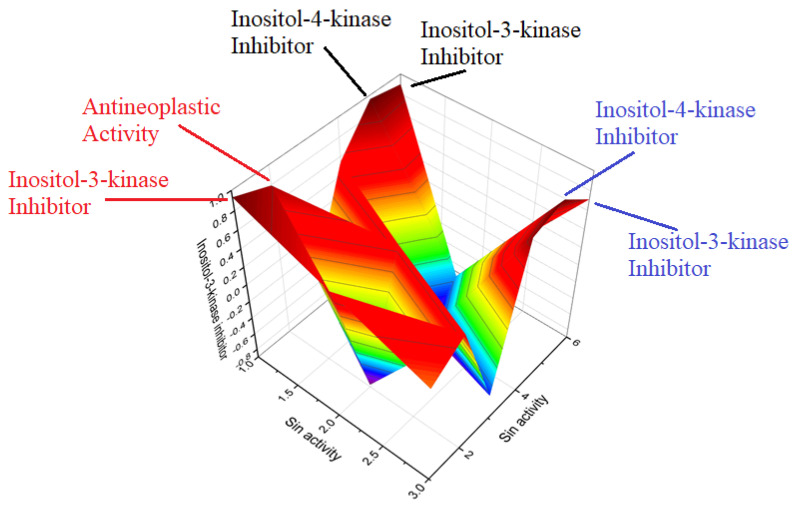
Three-dimensional graph illustrates the predicted and calculated activity as inositol-3-kinase inhibitors for steroids **7**, **8**, and **10** produced by the endophytic fungus *Talaromyces wortmannii*. The graph demonstrates a high confidence level of over 94% in the activity predictions. According to the data from Table 1, the dominant activity of steroids **7**, **8**, and **10** in the graph is shown in red. The steroid wortmanolone (**7**) is an antineoplastic and inositol-3-kinase inhibitor. The steroid wortmannin A (**8**) is an inositol-4-kinase inhibitor and inositol-3-kinase inhibitor of activity, and steroid secovironolide (**10**) is an inositol-4-kinase inhibitor and inositol-3-kinase inhibitor of activity. The activity text is in red for the steroid wortmanolone; in black is the activity of the steroid wortmannin A; and in blue is the activity of the steroid secovironolide. Red color—strong activity, blue color—no activity.

More recently, viridin (**2**) and the phytotoxin viridiol (**3**) were discovered in liquid cultures produced by the fungus *Gliocladium virens* [19]. Additionally, demethoxyviridin (**4**) and demethoxyviridiol (**5**) were isolated for the first time from an unidentified fungus [20]. The ash dieback-causing fungus *Hymenoscyphus pseudoalbidus* yielded furanosteroids (**17** and **18**) along with known compounds like viridiol (**6**) and demethoxyviridiol (**5**) [14,20,21]. Epoxyvirone (**11**) was detected in *Talaromyces* species (as depicted in Figure 5) and produced by the marine sponge-associated fungus *Talaromyces stipitatus* KUFA 0207 [22,23]. Ding and co-workers [24] reported the isolation of wortmannolone (**7**), wortmannin (**8**), 11-desacetoxywortmannin-17β-ol (**9**), secovironolide (**10**, represented by the 3D graph in Figure 6), epoxyvirone (**11**), wortmannin C (**13**), and 3-dihydrovirone (**12**) from the culture broth of the endophytic fungus *Talaromyces wortmannii* LGT-4, which was derived from the Chinese medicinal plant *Tripterygium wilfordii*.

Furthermore, a furanosteroid (**10**), along with viridiol (**3**), and **8**, **11**, and **13** were isolated from the soil fungus *Trichoderma virens*. Notably, 9-*epi*-viridiol (**23**) and viridiol (**2**) exhibited cytotoxicity against HeLa and KB cells, with IC_50_ values of 19 and 50 μg/mL, respectively [25].

Wortmannin (**8**) was initially isolated in 1957 by Brian and his colleagues from the broth of *Penicillium wortmanni* and subsequently from other fungi such as *P. funiculosum, Talaromyces wortmannii, Fusarium oxysporum, P. radicum*, and *Talaromyces* sp. [17,26,27]. Wortmannin and its derivatives, including compounds **7**, **8**, **9**, **13**, and **23**, exhibit antiproliferative properties and phosphatidylinositol 3-kinase activity [28,29,30,31]. Two furanosteroids, wortmannolone (**7**) and wortmannolol (**14**), were isolated from the fungal endophyte *Talaromyces* sp., which was obtained from *Tripterygium wilfordii* [31].

Another set of furanosteroids, namely 9-*epi*-viridiol (**15**) and viridiol (**3**), were isolated from *Trichoderma virens* [25]. Tricholumin A (**16**) possesses a unique carbon skeleton and is a highly transformed ergosterol derivative. It was isolated from the alga-endophytic fungus *Trichoderma asperellum*. Tricholumin A has demonstrated inhibitory effects against several pathogenic microbes, including *V. harveyi, V. splendidus*, and *Pseudoalteromonas citrea*. Additionally, it has displayed antifungal activity against *Glomerella cingulata* and has shown inhibition towards various marine phytoplankton species such as *Chattonella marina, Heterosigma akashiwo, Karlodinium veneficum*, and *Prorocentrum donghaiense* [35].

An unusual steroid compound, 1,11-epoxy-3-hydroxy-methylenecycloartan-28-oic acid (**17**), was isolated from an unidentified fungus [36]. Similar steroid structures have been identified in species belonging to the Agaricaceae family. Notably, a series of rare 1,11-epoxy lanostane-type triterpenoids called lepiotaprocerins A–F (**18**–**23**) were isolated from the fruiting bodies of the edible mushroom *Macrolepiota procera* collected in Poland. These compounds exhibited significant inhibitory effects on nitric oxide production, outperforming the positive control, L-NG-monomethyl arginine [37].

**Figure 7:**
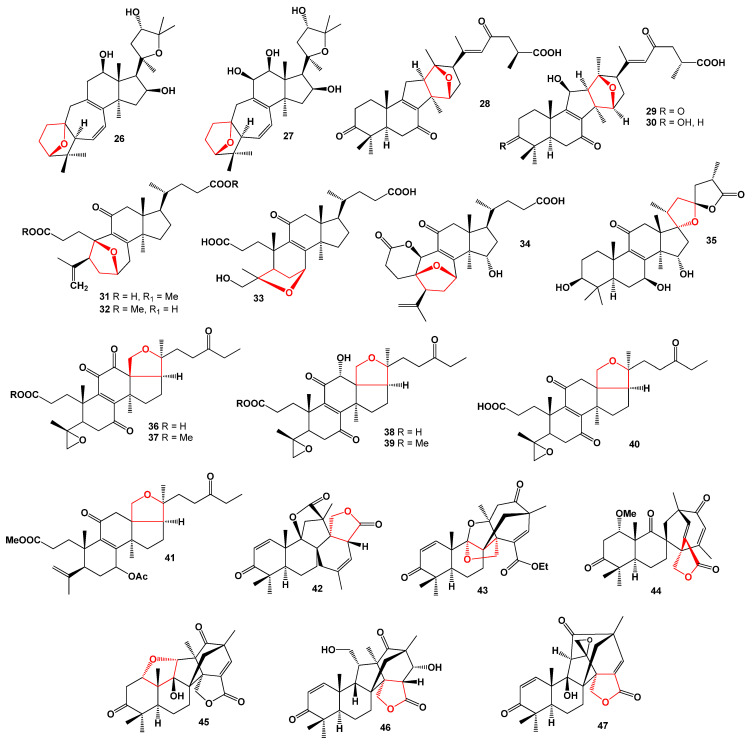
Furanosteroids produced by fungi and fungal endophytes.

Endophytic fungi originating from *Astragalus* species were found to catalyze the biotransformation of cycloastragenol, leading to the synthesis of an uncommon meroterpenoid compound **24** [48]. Additionally, through the utilization of an *Astragalus* endophyte called *Alternaria eureka* 1E1BL1, the biotransformation of cyclocephagenol, a novel cycloartane-type sapogenin with a tetrahydropyran unit, resulted in the formation of rare metabolites (**25**–**27**). The structures of these metabolites are depicted in Figure 7 [49].

Various *Ganoderma* species, used in traditional Asian medicine to prevent and treat diseases [50], have yielded intriguing seco-steroids (**28**–**41**), as depicted in Table 2. Extracts from the wood-decay fungus *Ganoderma applanatum* revealed the discovery of ganoapplanic acid A (**28**), ganodapplanoic acids A (**29**), and B (**30**). These rearranged lanotane-type triterpenoids possess a 6/6/5/6-fused tetracyclic structure, featuring an uncommon C-13/C-15 oxygen bridge moiety [51,52]. Furthermore, *G. cochlear* yielded cochlates A and B (**31** and **32**), isomeric compounds with a 3,4-seco-9,10-seco-9,19-cyclo skeleton [53], while fornicatin A (**33**), a 3,4-seco-trinortriterpenoid, was first isolated from the fruiting bodies of *G. fornicatum* [54].

**Figure 8:**
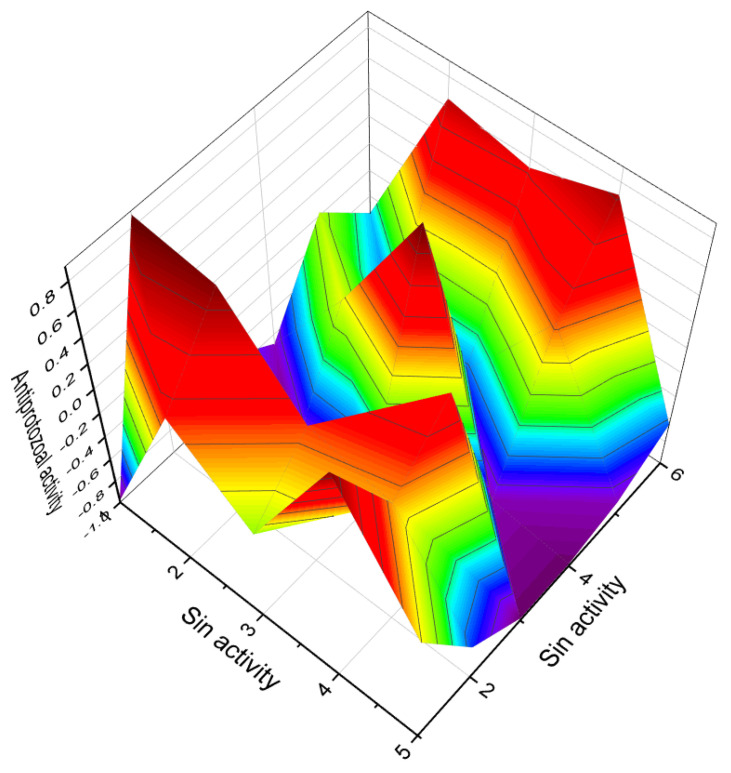
Three-dimensional graph illustrates the predicted and calculated antiprotozoal activity of steroids (**36**, **37**, **38**, **39**, and **40**) with a confidence level exceeding 95%. These steroids are produced by the medicinal mushroom *Ganoderma tropicum*.

An intriguingly rearranged hexanorlanostane, known as cochlate C (**34**), was obtained from the fruiting bodies of *Ganoderma cochlear* [55]. Additionally, *Ganoderma tropicum* and *G. boninense*, two medicinal mushrooms, yielded a steroid (**35**) and a series of secolanostanes (**36**–**41**, 3D graph, see Figure 8) with anti-plasmodial activity [56,57,58]. Furthermore, the fungal strain *Emericella* sp. TJ29, an endophyte derived from the root of *Hypericum perforatum*, produced several extraordinary meroterpenoids named emeridones A–F (**42**–**47**) [59].

**Figure 9:**
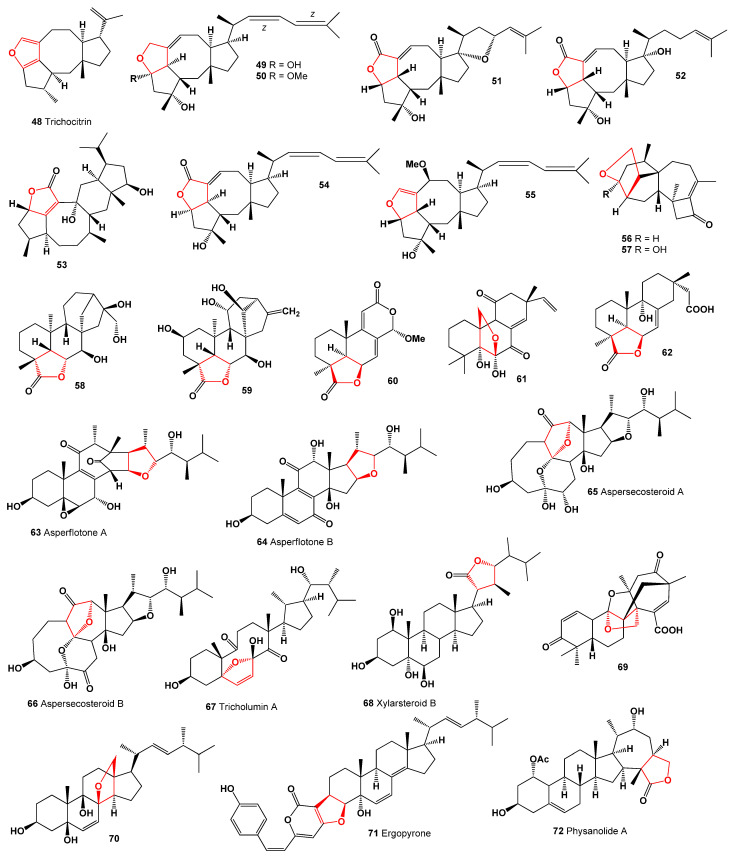
Steroids and isoprenoid lipids produced by fungi.

Trichocitrin (**48**), a diterpene, was extracted from the alga-endophytic fungus *Trichoderma citrinoviride*. Its structure is depicted in Figure 9, and the biological activity is outlined in Table 3. This compound represents the first furan-bearing fusicoccane diterpene derived from *Trichoderma* sp. and has shown inhibitory effects against *E. coli* [60]. The fungus *Aspergillus ustus*, isolated from the Mediterranean sponge *Suberites domuncula*, produced the sesterterpenoids ophiobolin-types **49** and **50** [61].

**Figure 10:**
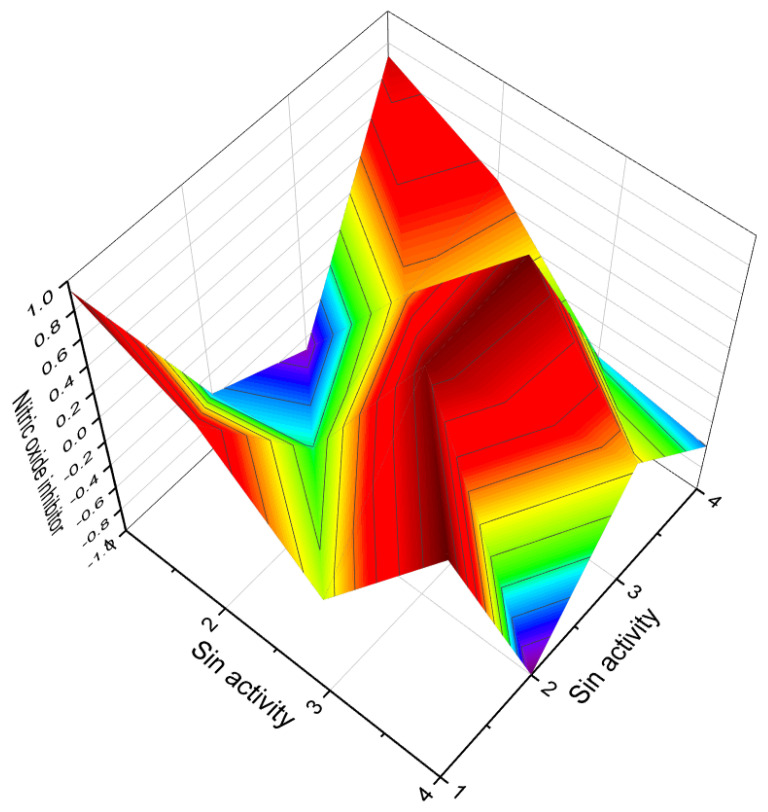
Three-dimensional graph illustrates the predicted and calculated activity of steroids (**49**, **50**, **54**, and **55**) as nitric oxide inhibitors, with a confidence level exceeding 90%.

Two phytotoxic sesterterpenoids, namely ophiobolin A lactone (**51**) and B (**52**), are synthesized by *Pseudomonas aeruginosa*, while compound **52** is also produced by the pathogenic fungi *Drechslera maydis* and *D. sorghicola* [62,63,64]. Additionally, the two mangrove fungi *Aspergillus terreus* H010 and *Lophiostoma bipolare* BCC25910 yielded compounds **53**–**55**; structures are shown in Figure 9, and a 3D graph is shown in Figure 10 [65,66].

Microbial transformation by the bacterial strain *Bacillus* sp. IMM-006 yielded two harziene-type diterpenoids, furanharzianones A (**56**) and B (**57**), featuring an unusual 4/7/5/6/5 ring system [67,68].

*Dictyophora rubrovolvata*, a saprophytic mushroom extensively cultivated in China, particularly in Guizhou Province, serves as a valuable source of two diterpenoids: 7,16,17-trihydroxy-19,6-kauranolide (**58**) and 2,7,11,14-tetrahydroxy-16-kauren-19,6-olide (**59**) [69]. Additionally, an *Acrostalagmus* fungus yielded LL-Z 1271a (**60**), a C16 terpenoid known for its antifungal properties [70,71]. Aspergilone A (**61**), an isopimarane compound, was isolated from a fungal species called *Epicoccum* [72], whereas xylarenolide (**62**), a pimarane derivative, has been identified in endophytic *Tubercularia* species [73] and certain *Xylaria* species [74,75].

From the solid culture of *Aspergillus flocculosus* 16D-1, two distinct steroids were isolated. The first one, asperflotone (**63**), is an unusual 8(14 → 15)-abeo-ergostane-type steroid. It possesses a unique ergosteroid structure characterized by a rearranged bicyclo [4.2.1]non-2-ene ring system, potentially resulting from α-ketol rearrangement during biosynthesis. Remarkably, both asperflotone and the second steroid, asperfloroid (**64**), exhibited inhibitory activity against IL-6 production in induced THP-1 cells [76]. In addition, the sponge-derived fungus *Aspergillus flocculosus* 16D-1 produced two other steroids, aspersecosteroids A (**65**) and B (**66**), which are 11(9→10)-abeo-5,10-secosteroids. Notably, both compounds demonstrated a potent inhibitory effect on the production of TNF-α and IL-6 [77].

Tricholumin A (**67**) was obtained from the alga-endophytic fungus *Trichoderma asperellum*. It retains cycle A, the final structural element of the original ergosterol, after undergoing deep oxidative transformations in the rest of the molecule, including the side chain fragment [35].

Xylarglycoside B (**68**), an antibacterial steroid, was isolated from the fungus *Xylaria* sp. KYJ-15, which was derived from the leaves of *Illigera celebica*. It displayed antibacterial activity against *Staphylococcus aureus* [78]. The endophytic fungus *Emericella variecolor* led to the isolation of emericellic acid (**69**), a meroterpenoid compound [79]. From the fruiting bodies of the mushroom *Stropharia rugosoannulata*, an unusual sterol with an unprecedented ether ring (**70**) was isolated. This mushroom is known as *saketsubatake* in Japanese and wine-cap *stropharia* in English [80]. Ergopyrone (**71**), an extraordinary styrylpyrone-fused ergosterol derivative, was isolated and structurally characterized from the mushroom *Gymnopilus orientispectabilis*. This steroid features a hexacyclic 6/5/6/6/6/5 skeleton that is formed via [3 + 2] cycloaddition between ergosterol and styrylpyrone precursors [81].

**Figure 11:**
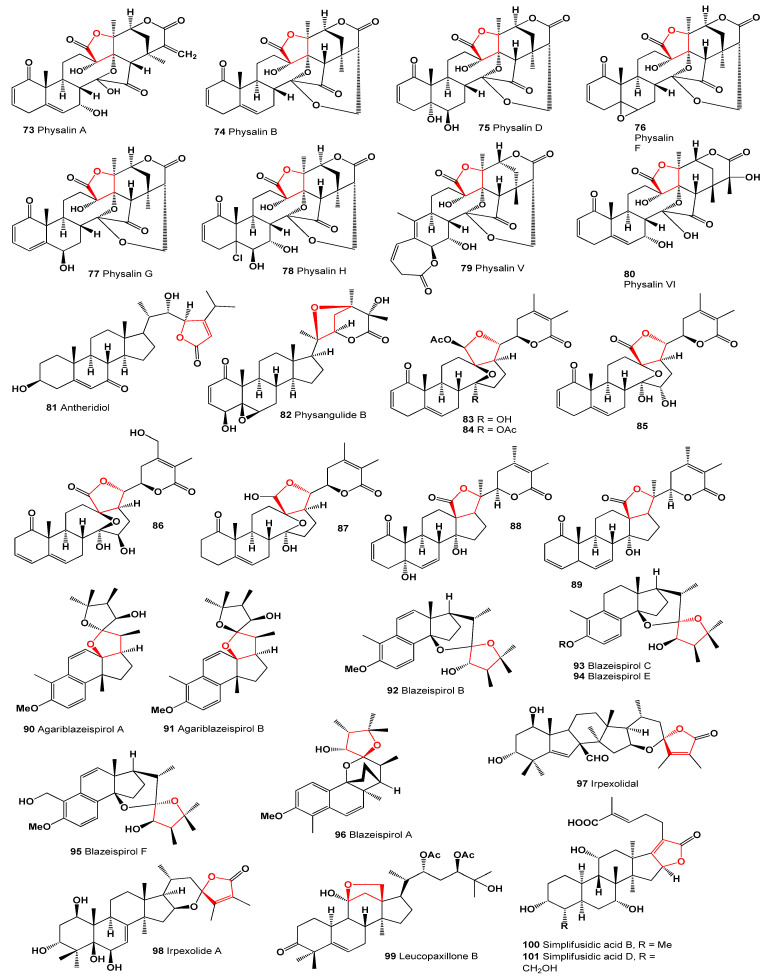
Steroids and meroterpenoids derived from fungal species.

*Physalis angulata* yielded several C28 steroids of the withasteroid family, including physanolide A (**72**) with an unprecedented skeleton containing a seven-membered ring and various physalins (**73**–**80**), as shown in Figure 11. These compounds displayed biological activity, as outlined in Table 4. Physalins B, D, and F exhibited potent cytotoxicity against multiple tumor cell lines, including KB, A431, HCT-8, PC-3, and ZR751, with EC_50_ values below 0.4 μM [82,83]. Antheridiol (**81**), the fungal sex hormone, was isolated from *Achlya bisexualis*, a water mold [84]. Physangulide B (**82**), a steroid, was identified in the calyxes of *Physalis angulata*, featuring an additional tetrahydrofuran ring in its structure [85].

Bioactive compounds **83**–**99**, known as withaphysalins, were isolated from *Physalis minima*. These compounds typically possess a hemiacetal or lactone linkage between C-18 and C-20, and they have demonstrated cytotoxic, antiproliferative, and anti-inflammatory activities [86,87].

**Figure 12:**
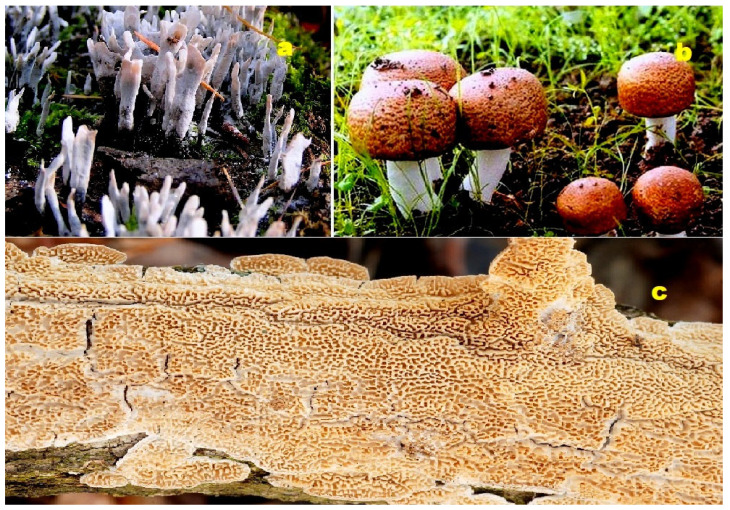
(**a**) *Xylaria* sp.: This ascomycete fungus typically thrives on dead wood and is known for producing steroids (**68**). The genus *Xylaria* comprises fungal endophytes associated with both vascular and non-vascular plants, and it serves as a valuable source of bioactive secondary metabolites. These metabolites include sesquiterpenoids, esters, alcohols, terpenoids, cytochalasins, mellein, alkaloids, polyketides, and aromatic compounds. Some of these compounds have demonstrated potential activity as herbicides, fungicides, insecticides, antibacterials, antimalarials, antifungals, or α-glucosidase inhibitors [94]. (**b**) *Agaricus blazei*: This medicinal fungus contains secosteroids (**90**–**95**). With significant commercial value, *Agaricus blazei* offers a wide range of health benefits. The mushrooms are rich in biologically active substances such as polysaccharides, lipids, sterols, proteins, vitamins B, C, and D, as well as phenolic compounds. Polysaccharides from *A. blazei* have been shown to possess immunoregulatory, anti-inflammatory, hepatoprotective, and antitumor properties. Extracts from this fungus have been used to treat diabetes and bacterial infections, exhibiting anticarcinogenic and antimutagenic effects [95,96]. (**c**) *Irpex lacteus*: This medicinal fungus serves as a source of triterpenoids (**97** and **98**). *Irpex lacteus*, a white rot fungus, is widely employed in bioremediation and food biotechnology due to its exceptional lignin-degrading capabilities. The fungus produces various extracellular enzymes, including lignin peroxidase, laccase, glucose oxidase, proteases, and α-galactosidase, involved in oxidative, hydrolytic, and lignocellulose degradation processes. *Irpex lacteus* readily oxidizes steroids, triterpenoids, alkanes, and cyclic ketones [97,98].

*Agaricus blazei* (a picture of this fungus is shown in Figure 12), also known as *‘Cogumelo do Sol*’ in Brazil or ‘*Himematsutake*’ in Japan, is a widely cultivated mushroom with medicinal uses.

**Figure 13:**
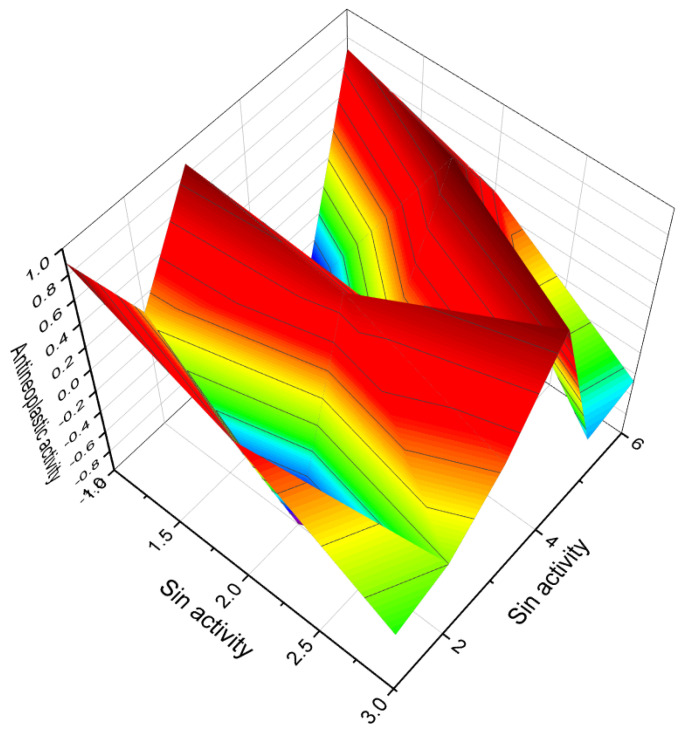
Three-dimensional graph illustrating the predicted and calculated antineoplastic activity of steroids (**74**, **75**, and **99**) with a confidence level exceeding 91%. These steroids are derived from meroterpenoids produced by the fungus *Physalis angulata*, while the steroid is sourced from the mushroom *Leucopaxillus gentianeus*.

It has been traditionally employed to treat various common ailments such as atherosclerosis, hepatitis, hyperlipidemia, diabetes, dermatitis, and cancer. The mushroom contains bioactive, highly oxygenated des-A-ergostane derivatives, including agariblazeispirols A (**90**) and B (**91**), as well as blazeispirols B (**92**), C (**93**), E (**94**), and F (**95**), which were isolated from cultured mycelia of *Agaricus blazei* [88,89]. Agariblazeispirols (**97**–**99**, 3D graph, see Figure 13) exhibited a moderate circumvention of drug resistance in mouse leukemia P388/VCR cells [89]. Additionally, blazeispirol A (**96**), featuring an unprecedented skeleton, has been isolated from the cultured mycelia of the same fungus [90].

A triterpenoid named irpexolidal (**97**) with an unprecedented carbon skeleton, along with its biogenetic-related compound irpexolide A (**98**) were isolated from the fruiting bodies of the medicinal fungus *Irpex lacteus* [91]. Study of the extract of the fruiting bodies of the mushroom *Leucopaxillus gentianeus*, allowed the isolation of minor cucurbitane triterpene, leucopaxillone B (**99**, the 3D graph is shown in Figure 13). The antiproliferative activity of the isolated triterpene was determined against the NCI-H460 human tumour cell line [92]. Simplifusidic acids B (**100**) and D (**101**), fusidane-type nortriterpenoids, were isolated from the marine-derived fungus *Simplicillium* sp. SCSIO 41513 [93].

## 3. Furanosteroids Derived from Plant Species

**Figure 14:**
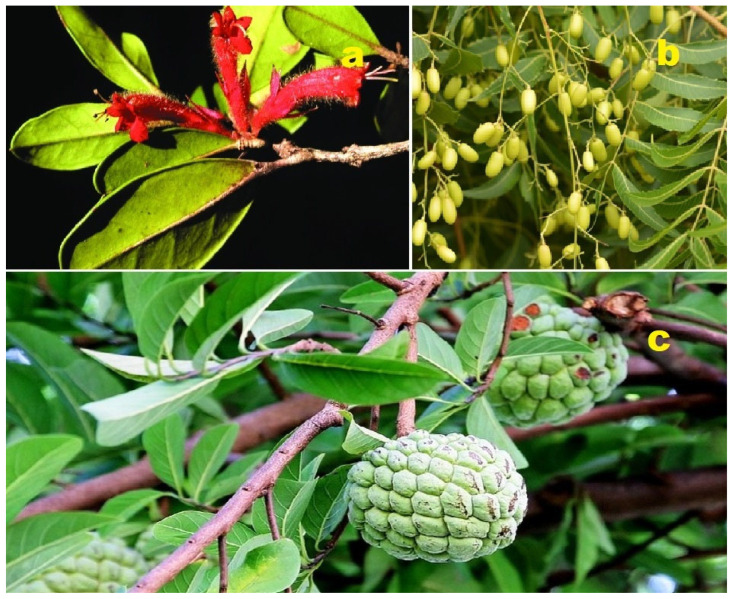
(**a**) *Malleastrum*: *Malleastrum* is a genus comprising over 20 species within the Meliaceae family. Native to Madagascar, the Comoros, and Aldabra, plants in the Meliaceae family are known to contain limonoids, terpenoids, alkaloids, flavonoids, and phenolic compounds as their primary chemical constituents. Many species within this family exhibit cytotoxic, antimicrobial, or antimalarial activity [107]. (**b**) *Azadirachta indica* (Neem): *Azadirachta indica*, commonly known as Neem, belongs to the Meliaceae family. The leaves of Neem are widely used in Chinese, Ayurvedic, and Unani medicines, particularly in the Indian subcontinent. Neem leaves have demonstrated antibacterial, anthelmintic, antiviral, and anticancer properties, and most notably, they act as an immunomodulatory agent [108]. (**c**) *Annona squamosa* (Sugar Apple): *Annona squamosa*, also known as sugar apple, is a member of the Annonaceae family. It has been traditionally used in Indian, Thai, and American medicine. The leaves of sugar apple are commonly used as a decoction to treat dysentery and urinary tract infections [109].

Furanosteroids derived from plant species have been extensively studied. Around 50 years ago, Indian scientists isolated a hexacyclic tetranortriterpenoid called vilasinin (**102**) from the green leaves of the neem tree (*Azadirachta indica*). Since then, numerous related metabolites have been discovered in various plant species [99]. 1,3-Diacetylvilasinin (**103**) has been reported from *Melia volkensii* [100], *Chisocheton paniculatus* [101], *Azadirachta indica* [102], as well as two African Turraea species, *Turraea holstii* and *T. parvifolia* [103]. Another compound, 1,3-diacetyl-12α-hydroxy-7-tigloylvilasinin (**104**), has been found in *Azadirachta indica* [104] and *Malleastrum antsingyense* (depicted in Figure 14) [105].

**Figure 15:**
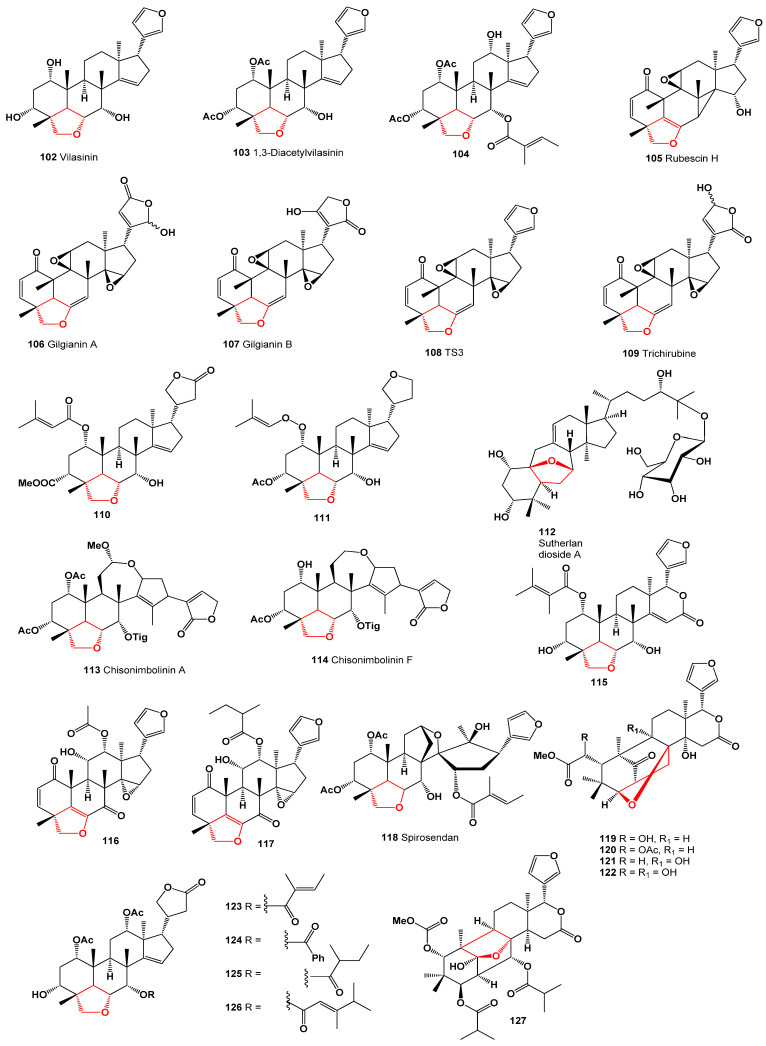
Steroids and meroterpenoids derived from plant species.

**Figure 16:**
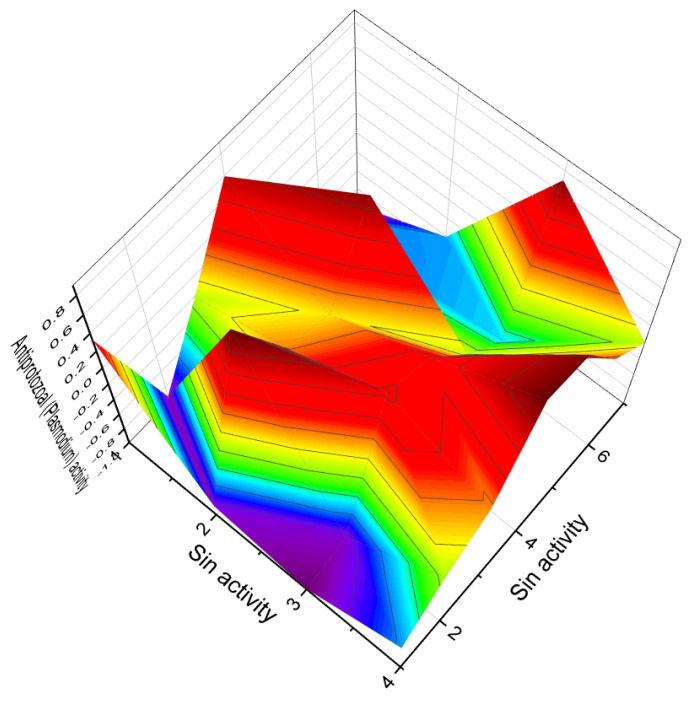
Three-dimensional graph illustrates the predicted and calculated antiprotozoal (*Plasmodium*) activity of the vilasinin-type limonoids (**106**, **107**, **108**, and **109**) with a confidence level exceeding 95%. These limonoids are derived from the tree *Trichilia gilgiana*, which is predominantly found in southern Nigeria and eastern Congo. In the Congo region, the extract of *Trichilia gilgiana* bark is utilized for its analgesic and stimulant properties. It has been traditionally used in traditional medicine to treat abdominal pain, chest pain, fever, and as a tonic. The juice of the young leaves is applied to circumcised wounds, while crushed leaves are added to drinking water for the treatment of respiratory diseases. Additionally, the juice of the leaves has demonstrated potent activity against the malarial *Plasmodium* [123,124,125].

The leaves of *Trichilia gilgiana*, extracted with CH_2_Cl_2_-MeOH, yielded vilasinin-type limonoids known as rubescin H (**105**), gilgianin A (**106**), gilgianin B (**107**), TS3 (**108**), and trichirubine A (**109**). Furanosteroids **105**, **106**, and **107** demonstrated potent anti-plasmodial activity alongside significant cytotoxicity. Compounds **108** and **109** exhibited the highest anti-plasmodial activity, with IC_50_ values of 1.1 and 1.3 μM, respectively [106]. The structures of compounds **105**, **106**, and **107** are shown in Figure 15, and their biological activity is detailed in Table 5. Furthermore, a 3D graph illustrating the activity of compounds **108** and **109** is displayed in Figure 16.

*Azadirachta indica*, belonging to the family Meliaceae, is commonly known as neem or Indian lilac. It is utilized for its antimalarial, anti-inflammatory, antipyretic, antitumor, and anthelmintic properties. The leaves of *Azadirachta indica* contain tetranortriterpenoids such as **110** and **111** [110].

*Sutherlandia frutescens*, a plant from the Fabaceae family native to South Africa, is commonly referred to as Cancer bush. It is renowned for its multifunctional medicinal uses, and infusions and decoctions of the plant are widely employed in South Africa for treating cancer, inflammation, viral infections, and gastrointestinal diseases. An unusual cycloartane glycoside called sutherlandioside A (**112**) has been isolated from the water-methanol fraction of the plant [111].

*Chisocheton paniculatus* twigs yielded two tetranortriterpenoids named chisonimbolinins A (**113**) and F (**114**) [112]. Furthermore, a triterpenoid **115** with insecticidal activity was isolated from a methanol extract of fresh leaves of *Azadirachta indica* [113].

*Walsura cochinchinensis* bark extract yielded two limonoids, walsucochinones B (**116**) and C (**117**). The ethyl acetate extract and walsucochinone C (**117**) displayed cytotoxic activity against MCF-7 human breast cancer cells [114]. *Melia toosendan* (Meliaceae) root bark provided spirosendan (**118**), a skeletal limonoid with a spiro-structure [115]. Additionally, an aqueous methanolic extract of Cedrela odorata leaves yielded four tetranortriterpenoids: cedrodorin (**119**), 6-acetoxycedrodorin (**120**), 6-deoxy-9*R*-hydroxycedrodorin (**121**), and 9*R*-hydroxycedrodorin (**122**) [116].

*Dysoxylum gaudichaudianum*, commonly known as ivory mahogany, yielded four tetranortriterpenoids named dysoxylins A–D (**123**–**126**), which exhibited potent antiviral activity against the respiratory syncytial virus [117]. *Xylocarpus rumphii* heartwood provided a triterpenoid derivative identified as xylorumphiins E (**127**) [118].

**Figure 17:**
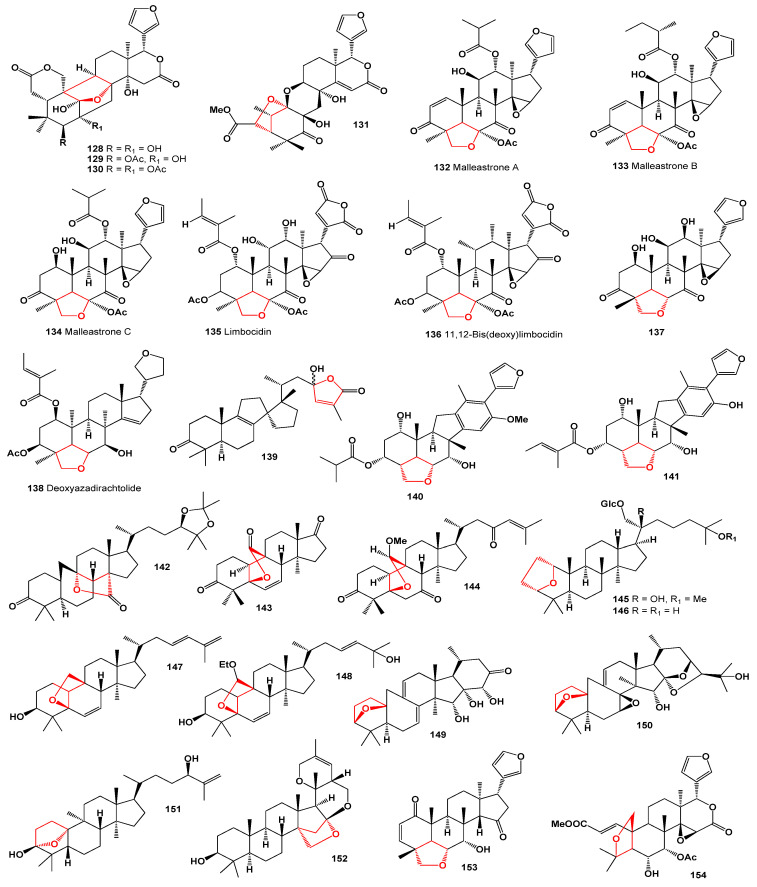
Bioactive Steroids and triterpenoids derived from plant species.

Stem bark extracts of *Khaya anthotheca* contained three limonoids: anthothecanolide (**128**), 3-O-acetylanthothecanolide (**129**), and 2,3-di-O-acetyl-anthothecanolide (**130**) [119]. The structure of compound **130** is depicted in Figure 17, and its biological activities are outlined in Table 6.

A tetranortriterpenoid called kokosanolide D (**131**) has been isolated from the methanol extract of fruit peels of *Lansium domesticum*, found in West Java, Indonesia [120]. Furthermore, an ethanol extract of a plant belonging to the *Malleastrum* genus yielded three steroids known as malleastrones A–C (**132**, **133**, and **134**), respectively. Compounds **133** and **134** exhibited antiproliferative activity against a range of cancer cell lines, with IC_50_ values ranging from 0.19 to 0.63 µM [121,122].

Limbocinin (**135**) and compound **136** were isolated from ethanolic extracts of neem seeds and the leaves of *Azadirachta indica* and *Annona squamosa* [126,127]. Both compounds exhibited antifungal activity [128].

Furanosteroids (**137** and **138**) have been isolated from plants belonging to the Meliaceae and Simaroubaceae families [129,130,131,132]. Furthermore, a rearranged lanostane (**139**) has been isolated from *Abies nephrolepis*, commonly known as Khingan fir [133].

**Figure 18:**
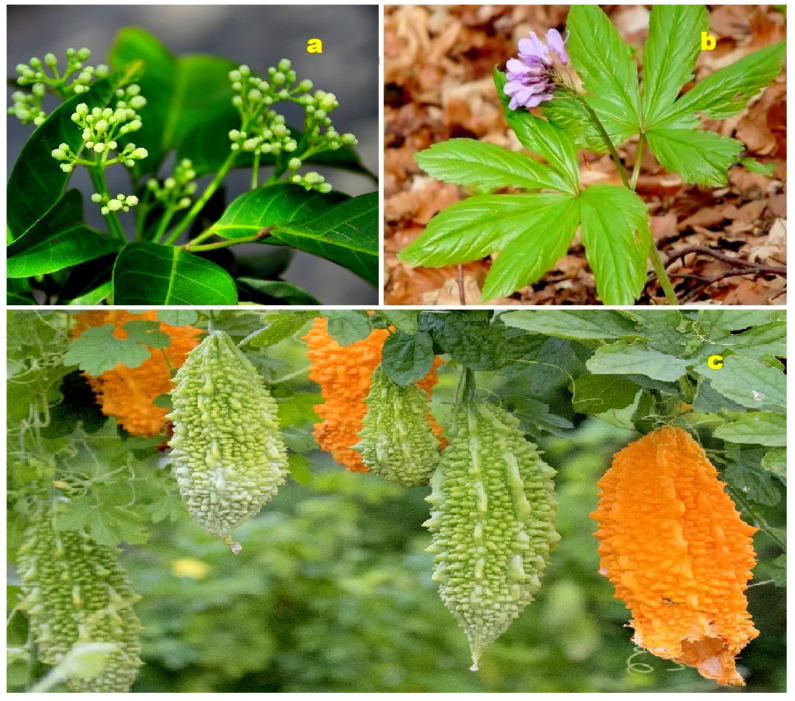
(**a**) *Walsura cochinchinensis*: This plant, belonging to the Meliaceae family, contains bioactive limonoids known as walsucochinoids A (**140**) and B (**141**). Medicinal plants from the *Walsura* genus, found in tropical areas of several Asian countries, are widely used in traditional medicine systems. Studies have identified over 200 compounds from ten species within this genus, including sesquiterpenoids, flavonoids, sterols, lignans, xanthones, and anthraquinones. Many of these compounds exhibit diverse properties such as cancer cell cytotoxicity, antimicrobial activity, antidiabetic effects, anti-inflammatory effects, antioxidant properties, antifeedant properties, antifertility effects, ichthyotoxic effects, and neuroprotective effects [134,135]. (**b**) *Gynostemma pentaphyllum*: Also known as Jiaogulan, *Gynostemma pentaphyllum* is a dioecious climbing vine from the Cucurbitaceae family. It is widely distributed in South and East Asia, as well as New Guinea. In Chinese medicine, it is commonly used to treat various conditions, including hepatitis, diabetes, and cardiovascular diseases. Extracts from *G. pentaphyllum* contain sterols, flavonoids, and polysaccharides that exhibit inhibitory activity against cancer cell proliferation. These extracts have demonstrated effects such as cell cycle arrest, apoptosis induction, inhibition of invasion and metastasis, inhibition of glycolysis, and immunomodulatory activity [136,137]. (**c**) *Momordica charantia*: The fruit of *M. charantia*, which contains anticancer steroids (**147** and **148**), has been consumed as food and used as medicine since ancient times. *M. charantia*, commonly known as bitter melon, holds a significant place in various systems of traditional medicine. It has been used to treat a wide range of conditions, including diabetes, abortive purposes, anthelmintic effects, contraception, dysmenorrhea, eczema, emmenagogue properties, antimalarial activity, lactagogue effects, gout, jaundice, abdominal pain, renal issues (stones), laxative effects, leprosy, leucorrhea, hemorrhoids, pneumonia, psoriasis, rheumatism, fever, and scabies [138,139].

Two limonoids with an aromatic ring D, walsucochinoids A (**140**) and B (**141**) were detected in the air-dried plant *Walsura cochinchinensis* (Figure 18 depicts a picture of this plant).

**Figure 19:**
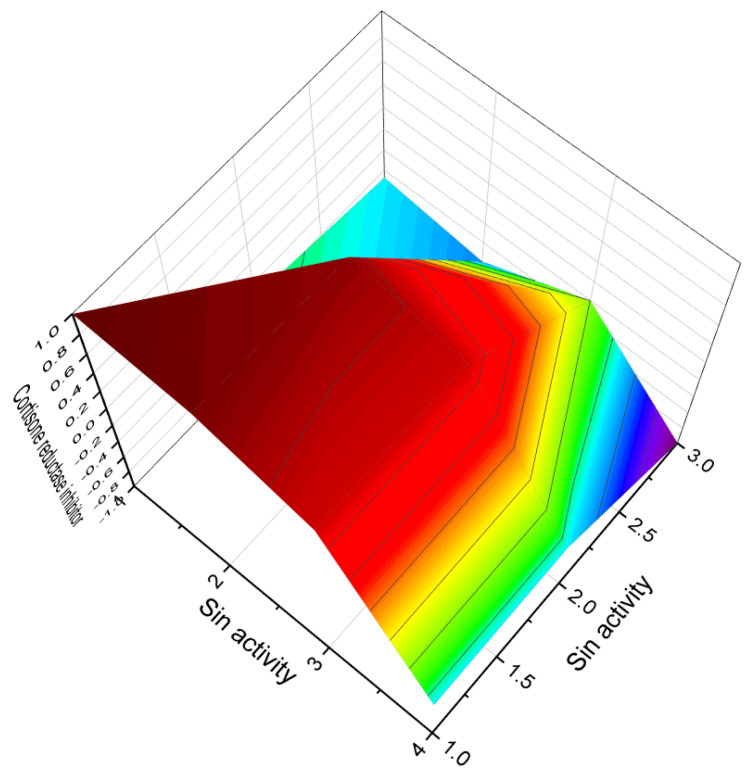
Three-dimensional graph illustrating the predicted and calculated activity of steroids (**140**, **141**, and **142**) as cortisone reductase inhibitors with over 90% confidence. These steroids include the limonoids with an aromatic ring D (**140** and **141**) found in the leaves of the *Walsura cochinchinensis* plant, as well as the triterpenoid (**142**) found in the leaves of *Caloncoba glauca*.

From the leaves of *Caloncoba glauca*, a triterpenoid called caloncobalactone C (**142**) was isolated. It exhibited inhibitory activity against human and mouse 11β-hydroxysteroid dehydrogenase types 1 and 2 [140,141]. The 3D graph illustrating its structure is presented in Figure 19.

Within the EtOAc and n-BuOH extracts of the vines and leaves of *Momordica aurantia*, two steroids, octanorcucurbitacin (**143**) and kuguacin I (**144**), were discovered. These compounds demonstrated anti-HIV-1 activities in vitro [142]. Triterpenes gypensapogenin H (**145**) and I (**146**) were isolated from the hydrolyzate of the total saponin extract obtained from the dioecious herbaceous climber *Gynostemma pentaphyllum*, which is widely distributed in South and East Asia [143].

Two cucurbitane-type triterpenoids, namely (23*E*)-5β,19-epoxycucurbita-6,23,25-triene-3β-ol (**147**) and (19*R*,23*E*)-5β,19-epoxy-19-ethoxycucurbita-6,23-diene-3β,25-diol (**148**), have been isolated from the fruit of *Momordica charantia*. These compounds exhibited weak cytotoxic activity against cancer cell lines including MCF-7, HepG2, Du145, Colon205, and HL-60 [144].

The roots of Cimicifuga heracleifolia, a plant included in the Chinese Pharmacopoeia and used in traditional medicine in China for centuries, contain two unusual ring A cracking 9,19-cycloartane triterpenes (**149**) and (**150**) [145]. The dried fruit of Vitex negundo yielded the 3,10-epoxide (**151**) with antitumor activity [146].

From the seeds of *Hovenia trichocarpa*, two saponins named hoduloside XI and hoduloside XII were isolated. Both compounds shared the genin 20,26-epoxy-pseudojujubogenin (**152**) and displayed inhibitive activities against human cancer cell lines HL-60 and K562 [147]. In addition to these steroids, a furanosteroid named ceramicine J (**153**) was discovered in the hexane layer of *Chisocheton ceramicus* bark extract. This compound exhibited dose-dependent, moderate cytotoxicity against the HL-60 cell line [148]. Furthermore, a secosteroid called 6-O-deacetylseverinolide (**154**) was identified in the stem barks of *Atalantia buxifolia* extract [36].

**Figure 20:**
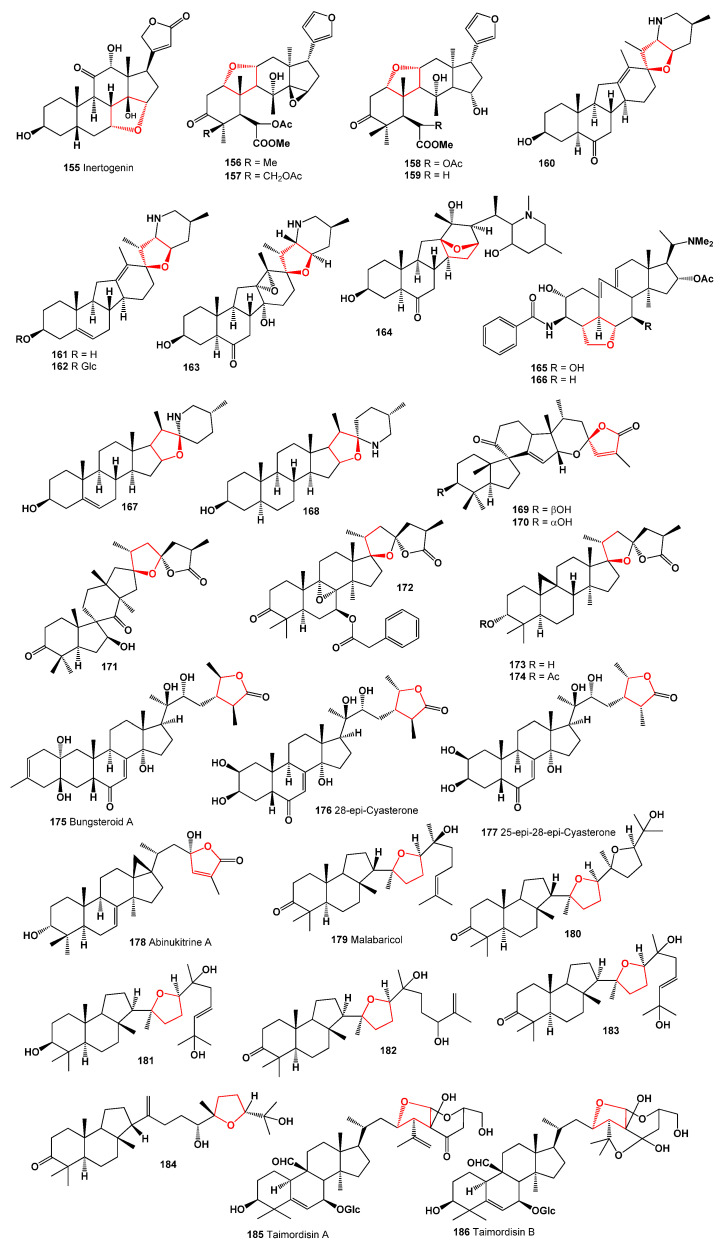
Steroids, di-, and triterpenoids derived from plant species.

The steroid inertogenin (**155**) is a compound containing a rare 7,15-tetrahydrofuran group. It is found in the leaves of *Strophanthus amboensis*, an erect deciduous shrub harvested for medical use in Southwest Africa [149]. The structure of inertogenin is depicted in Figure 20, and its biological activity is shown in Table 7. The leaves of *Toona ciliata* var. *yunnanensis* yielded several seco steroids: tooonayunnanins F (**156**), G (**157**), J (**158**), and K (**159**) [150].

**Figure 21:**
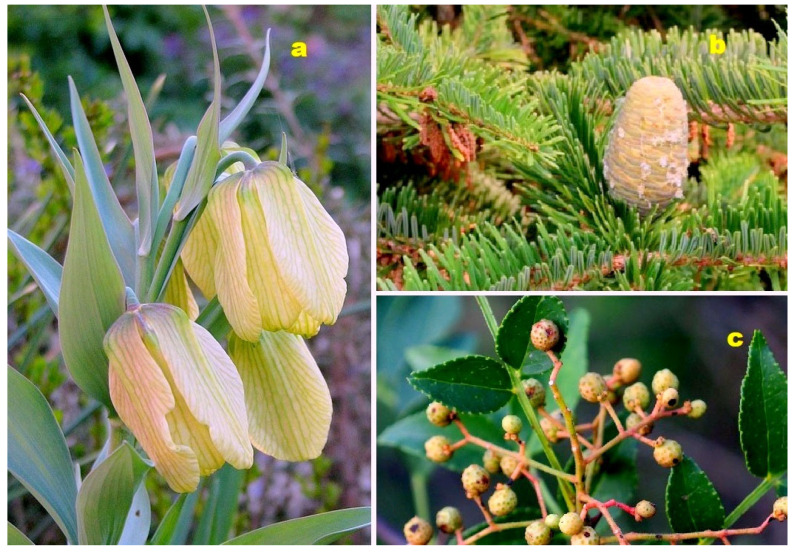
(**a**) The plant *Fritillaria pallidiflora* is a source of unusual steroids (**160**–**164**). *F. pallidiflora*, also known as Siberian hazel grouse, is a species that was initially misnamed as it does not grow wild in Siberia. It was discovered in 1857 in the western regions of the Himalayas and Asia Minor. The plant is known for its medicinal uses, and an infusion of dried chopped onion from this plant is commonly used orally to treat cough, bronchitis, pneumonia, febrile illnesses, and abscesses [162,163]. Shaanxi fir (**b**) is a tree that grows in Gansu, Hubei, and Sichuan. *Abies chensiensis*, a species of Shaanxi fir, produces unusual triterpenoids (**169** and **170**). The essential oil of Shaanxi fir, as well as Siberian fir (*Abies sibirica*), has a pleasant, fresh pine aroma and contains bornyl acetate, which contributes to its soothing, balancing, and anti-inflammatory effects. The oil is used to relieve anger, promote contentment, alleviate intolerance in toxic relationships, promote self-connection, and foster a fearless attitude [164,165]. The pericarps of *Zanthoxylum bungeanum* (**c**) produce an unusual steroid (**175**). Extracts from *Z. bungeanum* fruit are widely used in the cosmetics industry to produce creams. Various parts of this plant, including the fruit, stems, leaves, and bark, have been utilized in local medical systems to treat fever, stomach pain, toothache, and inflammation [166,167].

**Figure 22:**
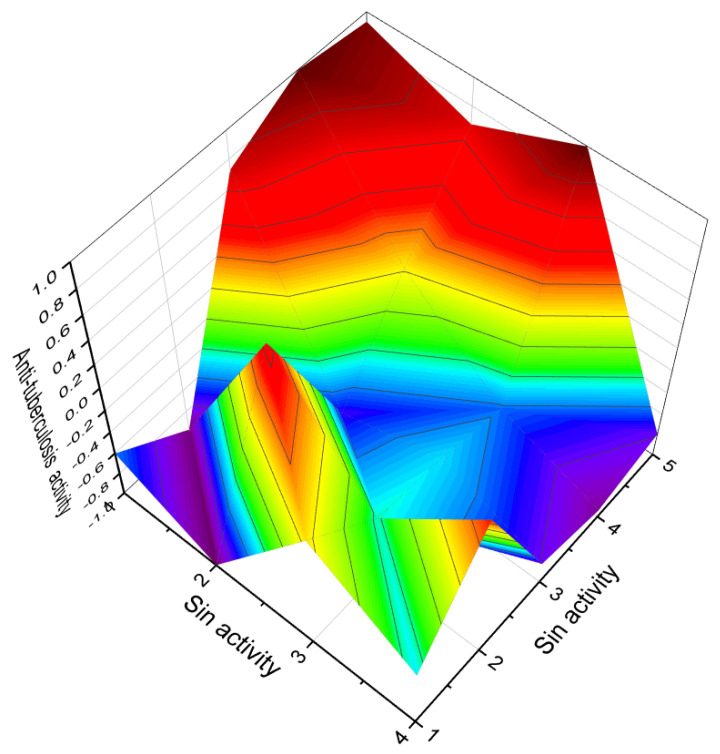
Three-dimensional graph illustrating the predicted and calculated anti-tuberculosis activity of steroids (**160**, **161**, **162**, **163**, and **164**) with a confidence level exceeding 90%. These steroids include the unusual steroidal jervinine-type alkaloids (**160**–**163**) derived from the bulbs of *Fritillaria pallidiflora*, as well as alkaloid (**164**) obtained from the bulbs of *Fritillaria puqiensis*. Notably, these compounds possess the rare property of exhibiting anti-tuberculosis activity. The genus *Fritillaria* has been widely used worldwide for medicinal and culinary purposes. For over 2000 years, decoctions made from the bulbs of various Fritillaria species have been utilized in traditional Chinese medicine to address diverse ailments such as asthma, pharyngitis, bronchitis, coughs, goiter, and hemoptysis. Additionally, it has been employed as an expectorant and antitussive agent [175,176].

*Fritillaria pallidiflora* bulbs are a source of unique jervinine-type alkaloids [151]. Peimissine (**160**), cycloparnine (**161**), and cycloposine (**162**) were isolated from the bulbs of *F. pallidiflora*, as well as the isosteroidal alkaloid yibeinone A (**163**) [152]. Puqienine F (**164**), a veratramine alkaloid with a 12,16-epoxy ring, was isolated from the bulbs of *Fritillaria puqiensis* [153]. A picture of *Fritillaria pallidiflora* is shown in Figure 21, and the 3D graph representing the activity of puqienine F (**164**) can be found in Figure 22.

From *Buxus hyrcana*, collected in Iran, two steroidal alkaloids were isolated: (+)-O6-buxafurandiene (**165**) and (+)-7-deoxy-O6-buxafurandiene (**166**). These compounds belong to the rare class of *Buxus* alkaloids with a tetrahydrofuran ring incorporated into their structures. Furthermore, they exhibited acetylcholinesterase enzyme inhibitory activity [154].

Steroidal alkaloids, solasodine (**167**), and tomatidine (**168**) were isolated from the aerial parts of *Solanum leucocarpum*, a plant belonging to the Solanaceae family. The collection was made at the regional natural park Ucumarí in Colombia. Both alkaloids have demonstrated biological activity. Solasodine exhibits DNA-damaging activity, while tomatidine displays activity through DNA topoisomerase II inhibition [155].

Two unique abeo-steroids, spirochensilides A (**169**) and B (**170**), were isolated from *Abies chensiensis*. These compounds represent the first example of triterpenoids with a distinctive 8,10-cyclo-9,10-seco and methyl-rearranged carbon skeleton [156]. *Abies faxoniana*, an endemic plant found in several provinces in China, serves as the source of lanostane and cycloartane derivatives A1/A2, **171**–**174**, which feature epimeric spiro-side chains [157].

Bungsteroid A (**175**), possessing an unreported carbon skeleton, was isolated from the pericarps of *Zanthoxylum bungeanum*. It represents a C34 steroid analogue with a unique 6/6/6/6/5-fused pentacyclic skeleton. This compound exhibited antiproliferative effects against HepG2, MCF-7, and HeLa cell lines, with IC_50_ values of 56.3, 64.2, and 74.2 μM, respectively [158].

The roots and stems of *Cyathula officinalis*, also known as *Cyathula Root* or *Radix Cyathula*, yielded two cyasterone stereoisomers: 28-*epi*-cyasterone (**176**) and 25-*epi*-28-*epi*-cyasterone (**177**) [159]. The biologically active plant steroid, 28-*epi*-cyasterone (**176**), has also been found in *Eriophyton wallchii* [160] and fronds of the fern *Microsorum scolopendria* [161].

Triterpenoids with medicinal properties have been identified from various plant sources. Abinukitrine A (**178**), a triterpenoid, was isolated from *Abies nukiangensis* extracts and exhibited a potent anti-hepatitis C virus (HCV) effect [62]. Malabaricol (**179**), another triterpenoid, was first reported by Indian chemists from the National Chemical Laboratory in 1967, isolated from the tropical tree *Ailanthus malabarica* [168]. Subsequently, malabaricol (**179**) and **180** were also found in the heartwood of *Ailanthus excelsa* [169]. Malabaricol and its derivatives [170] possess antibacterial effects, supporting their traditional use in folk medicine [171]. *Ailanthus triphysa* is a source of ailanthusins F (**181**) and G (**182**) [172], while two other derivatives, **183** and **184**, were discovered in the leaves of *Caloncoba echinata* [173].

Furthermore, triterpene glycosides called taimordisins A (**185**) and B (**186**) were isolated from the fresh fruits of Taiwanese *Momordica charantia* [174]. Although these compounds demonstrate favorable anti-inflammatory activity, they do not exhibit any anti-cancer properties and have been determined to be safe for use.

## 4. Furanosteroids Derived from Marine Sources

Furanosteroids, a fascinating group of natural compounds, are derived from marine sources and are produced by various marine invertebrates, including cyanobacteria, fungal endophytes associated with algae, sponges, soft corals, or molluscs [2,3,4,5,6,7,8].

**Figure 23:**
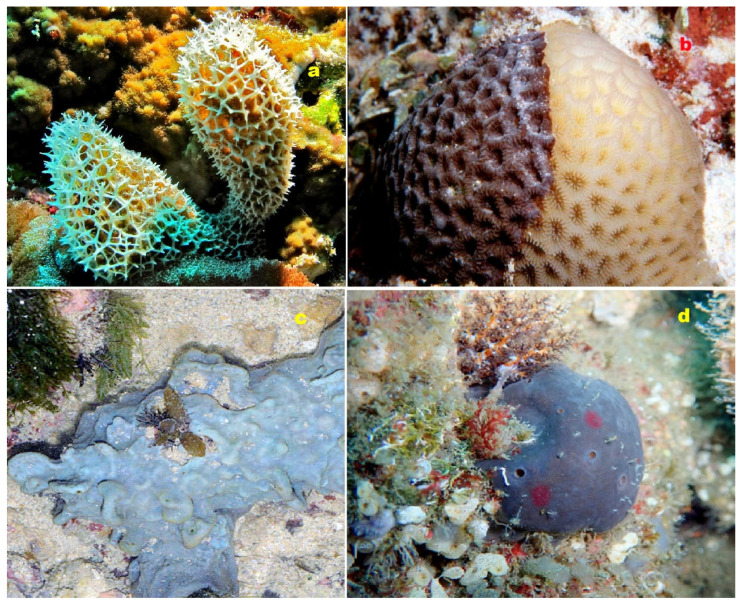
(**a**) The marine sponge belonging to the genus *Dysidea* is the origin of dysideasterols F (**188**) and G (**189**). (**b**) The Okinawan marine sponge *Terpios hoshinota* produces the norsteroid nakiterpiosin (**193**). (**c**) The marine sponge *Lendenfeldia frondosa*, found in the Solomon Islands, contains epihomoscalaralactone IIA (**204**). (**d**) The Vietnamese marine sponge *Penares* sp. serves as the source of lanostanes (**197** and **198**). These diverse marine organisms contribute to the production of biologically active compounds with potential pharmacological applications.

**Figure 24:**
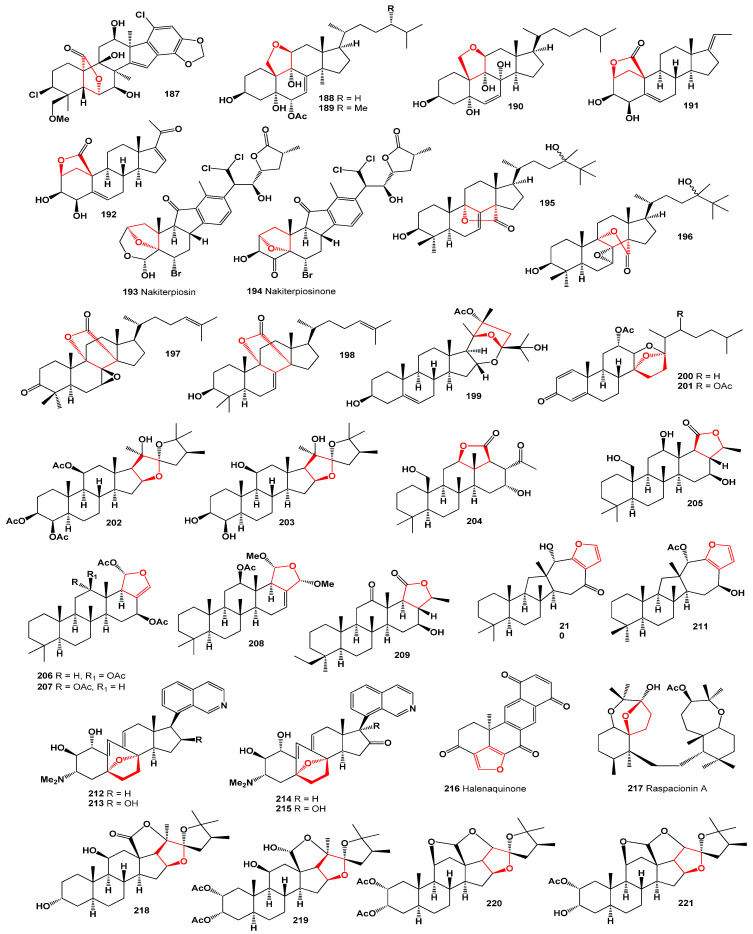
Furanosteroids and isoprenoid lipids derived from marine sources.

Merosterol A (**187**), a cyanobacterial cytotoxin found in *Scytonema* sp. PCC 10023, has been exhibited against HeLa cells with IC_50_ values of 1.8 µM [177]. In an active organic extract obtained from an Okinawan marine sponge of the genus *Dysidea* (depicted in Figure 23), two polyoxygenated steroids, dysideasterols F (**188**) and G (**189**), were identified. These compounds demonstrated a similar cytotoxic effect, with IC_50_ values of 0.15 and 0.3 µM, respectively, against human epidermoid carcinoma A431 cells [178]. Additionally, a toxic polyoxygenated steroid named cholest-6-en-11β,19-epoxy-3β,5α,8α,9α-tetrol (**190**) has been isolated from the sponge *Dysidea tupha* [179]. Furthermore, a sponge species, *Strongylophora* sp., yielded furano-pregnanes, namely 3,4-dihydroxypregna-5,17-diene-10,2-carbolactone (**191**, whose structure is shown in Figure 24) and 3,4-dihydroxypregna-5,15-dien-20-one-10,2-carbolactone (**192**) [180].

Nakiterpiosin (**193**) and nakiterpiosinone (**194**, whose structures are shown in Figure 24), which are halogenated and rearranged norsteroids, were isolated from the Okinawan marine sponge *Terpios hoshinota*. These compounds have demonstrated cytotoxicity against murine P388 leukemia cells [181].

Unusual steroids known as erylosides T (**195**) and U (**196**), derived from the sea sponge *Erylus goffrilleri*, contain novel genins [182]. Lanostanes (**197** and **198**), whose structures are depicted in Figure 24 and whose biological activity is presented in Table 8, were obtained from the marine sponge *Penares* sp., found in Vietnamese waters [183]. These discoveries highlight the diverse range of bioactive compounds originating from marine sources.

A spiroketal steroid, **199**, was obtained from a collection of *Gorgonella umbraculum* found in the Indian Ocean off the Tuticorin coast [184]. From a Japanese octocoral species, *Dendronephthya* sp., two secosteroids named isogosterones A (**200**) and B (**201**) were isolated. These compounds share common structures characterized as 12α-acetoxy-13,17-seco-cholesta-1,4-dien-3-ones with hemiacetal functionality. Isogosterones A and B exhibited inhibition of larval settlement in the barnacle *Balanus amphitrite*, with an EC_50_ value of 0.2 μM [185].

Furthermore, two steroids were isolated from the marine sponge *Isis hippuris*, also known as sea bamboo, collected from the Andaman Islands, India. These compounds are 3,11-diacetylhippurin-1 (**202**) and 22-*epi*-hippuri-stanol (**203**) [186]. Additionally, the marine sponge *Lendenfeldia frondosa*, collected from the Solomon Islands, yielded epihomoscalaralactone IIA (**204**) [187], while another marine sponge, *Phyllospongia dendyi,* from the Indian Ocean contained homoscalaralactone I1 B (**205**) [188]. Bioactive scalaranes (**206**–**208**) have been identified in the bio-toxic extracts of the marine sponge *Hyrtios erecta* [189]. Phyllofolactone A (**209**), isolated from the marine sponge *Phyllospongia* (syn. *Carteriospongia*) *foliascens* found in the South China Sea, has demonstrated cytotoxicity against P-388 cells [190].

**Figure 25:**
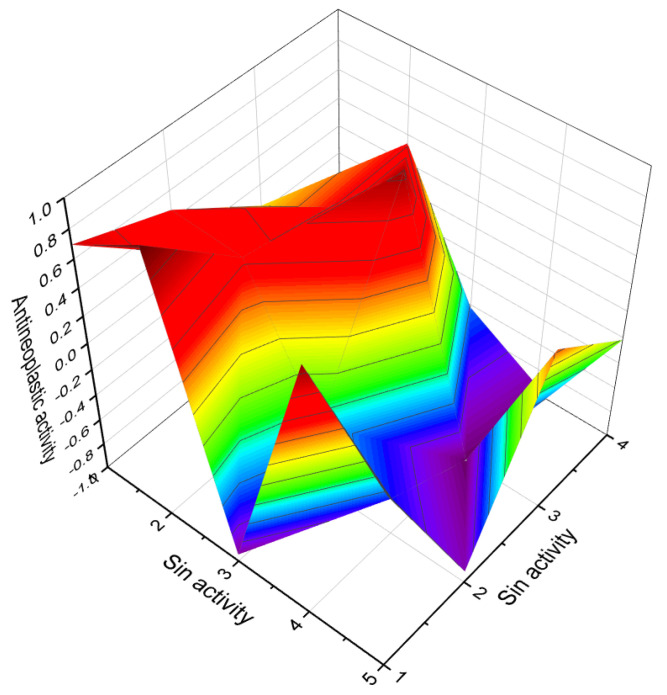
Three-dimensional graph illustrates the predicted and calculated antineoplastic activity of steroids (**210**, **218**, **219**, **220**, and **221**) with a confidence level exceeding 92%. These cytotoxic, highly polyoxygenated steroids are isolated from marine organisms inhabiting the waters surrounding Taiwan. Specifically, a metabolite (**210**) is found in the marine sponge *Hippospongia* sp., while the bamboo coral *Isis hippuris* contains compounds (**218**–**221**). These discoveries highlight the potential of marine organisms to provide valuable bioactive compounds with antineoplastic properties.

The marine sponge *Hippospongia* sp. collected from Taitung, Taiwan, serves as the source of a cytotoxic metabolite called hippospongide A (**210**) [191], as depicted in the 3D graph shown in Figure 25. Furthermore, the same compound, named salmahyrtisol A (**211**), has been isolated from the marine sponge *Hyrtios erecta* and exhibits significant cytotoxicity against murine leukemia (P-388), A-549, and HT-29 human cancer cells [192]. These findings underscore the potential of marine sponges as a valuable source of bioactive compounds with cytotoxic properties.

Steroidal anticancer alkaloids known as cortistatins A (**212**), B (**213**), C (**214**), and D (**215**) have been isolated from the marine sponge *Corticium simplex*. These alkaloids possess a unique 9 (10–19)-abeo-androstane and isoquinoline skeleton and have demonstrated the ability to inhibit the proliferation of human umbilical vein endothelial cells with high selectivity [193].

**Figure 26:**
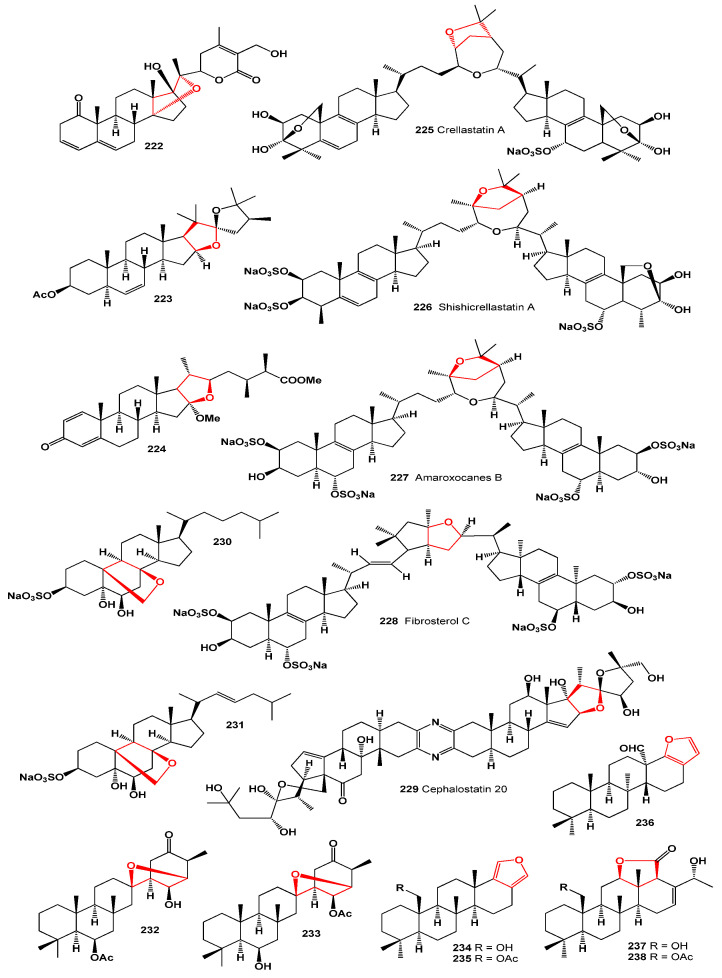
Furanosteroids, dimeric and isoprenoid lipids derived from marine sources.

Metabolites containing the core structure of viridin have been extracted from marine invertebrates. Over 40 years ago, a research group led by Paul Scheuer at the University of Hawaii at Mānoa isolated demethoxyviridin (**4**) and its furano-quinone analogue called halenaquinone (**216**) from the marine sponge *Xestospongia exigua* [194]. Raspacionin A (**217**), a triterpenoid, was obtained from the red sponge *Raspaciona aculeata* found in the Mediterranean Sea. This compound exhibited cytotoxicity against the MCF-7 tumor cell line, with an IC_50_ value of 4 μM [195,196,197]. Highly polyoxygenated steroids (**218**–**221**) containing three or four additional tetrahydrofuran fragments were discovered in the bamboo coral *Isis hippuris*, collected from the Southeast coast of Taiwan. These steroids displayed cytotoxic activity against Hep G2, Hep 3B, A549, MCF-7, and MDAMB-231 cells [198]. Additionally, two steroids (**222** and **223**), whose structures are depicted in Figure 26 and whose biological activity is presented in Table 9, were isolated from the soft coral *Sarcophyton crassocaula* found in the Indian Ocean [199]. Furthermore, the cytotoxic compound sinubrasone B (**224**) was identified in the reef soft coral *Sinularia brassica* [200].

Crellastatin A (**225**), a unique nonsymmetric dimeric steroid, was isolated from the marine sponge *Crella* sp. found on Vanuatu Island. This compound showcases an unprecedented connection through its side chains. Crellastatin A has been shown to possess significant in vitro cytotoxic activity against NSCLC-N6 cells, with an IC_50_ value of 0.5 μM [201]. This discovery underscores the potential of marine organisms to produce novel bioactive compounds with cytotoxic properties.

A similar dimeric steroid derivative, shishicrellastatin A (**226**), has been isolated from the marine sponge *Crella (Yvesia) spinulata*. This compound functions as a cathepsin B inhibitor, exhibiting an IC_50_ value of 8 μg/mL [202]. Another dimeric steroid, amaroxocane B (**227**), was discovered in the Caribbean coral reef sponge *Phorbas amaranthus* collected off Key Largo, Florida. It has shown effectiveness as an antifeedant [203]. Additionally, the same sterol dimer, hamigerol B (**227**), has been identified in the extract of the Mediterranean sponge *Hamigera hamigera* [204].

**Figure 27:**
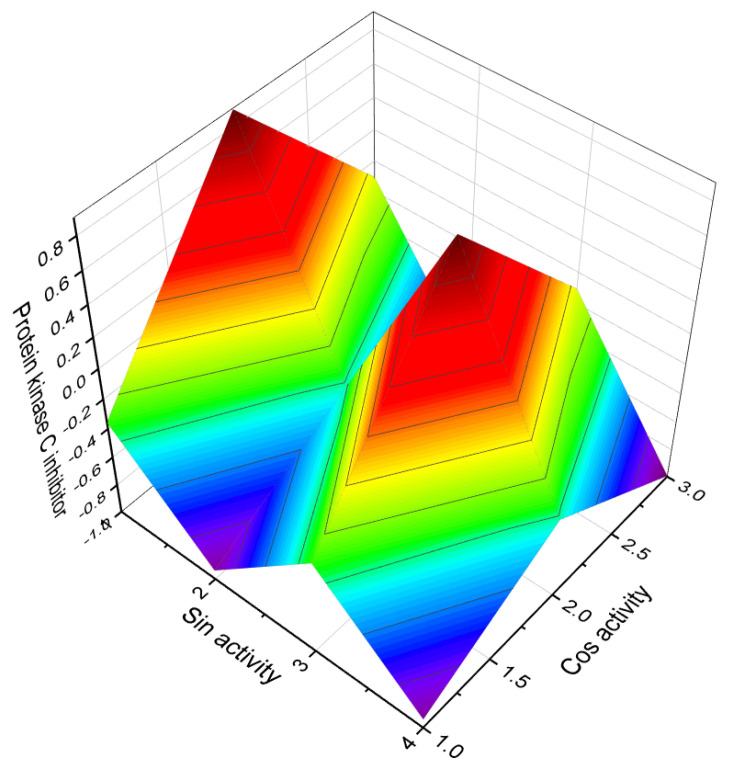
Three-dimensional graph presents the predicted and calculated activity of the steroid (**228**) with a confidence level exceeding 92%. This steroid is recognized as a protein kinase C inhibitor and exhibits potential as an anticancer and antiviral agent. It is a sulfated sterol dimer found in the marine sponge *Lissodendoryx (Acanthodoryx) fibrosa*, which belongs to the family Coelosphaeridae. This marine sponge is commonly found in the oceanic waters of the Philippines. The discovery of this compound highlights its promising bioactive properties derived from marine sources.

**Figure 28:**
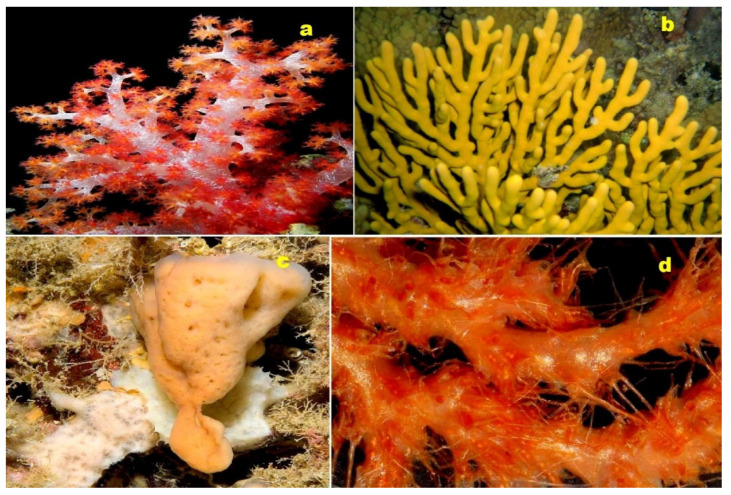
(**a**) The octocoral *Dendronephthya* sp. from the Sea of Japan produces secosteroids, namely isogosterones A (**200**) and B (**201**). (**b**) The marine sponge *Isis hippuris* serves as a source of steroids (**202**) and (**203**). (**c**) The marine sponge *Corticium simplex* produces steroidal alkaloids (**212**–**215**). (**d**) The marine worm *Cephalodiscus gilchristi* synthesizes a unique steroidal alkaloid (**229**). These diverse marine organisms contribute to the production of bioactive compounds with potential pharmacological applications.

Furthermore, a sulfated sterol dimer named fibrosterol C (**228**), obtained from *Lissodendoryx (Acanthodoryx) fibrosa* collected in the Philippines, has been found to inhibit protein kinase Cζ with an IC_50_ value of 5.6 μM [205]. The 3D graph illustrating its activity is shown in Figure 27. Cephalostatin 20 (**229**), a member of the cephalostatin family known for its anticancer properties, has been isolated as a minor component of extracts from the marine worm *Cephalodiscus gilchristi* [206]. A picture of this sea sponge is displayed in Figure 28.

An undescribed marine sponge from the genus *Euryspongia* serves as the source of sulfated steroids, namely eurysterols A (**230**) and B (**231**). Eurysterol A exhibits cytotoxicity against human colon carcinoma (HCT-116) cells, with an IC_50_ value of 0.3 μM. It also demonstrates antifungal activity against amphotericin-B-resistant *Candida albicans* [207]. Two sesterterpenoids, oxaspirosuberitenone (**232**) and isooxaspirosuberitenone (**233**), have been isolated from the marine sponge *Phorbas areolatus*. These compounds exhibit significant growth-inhibitory effects against A549, HepG2, HT-29, and MCF-7 tumor cell lines [208].

Furthermore, 12-dehydroxy-16-deacetoxy-22-hydroxyscalarafuran (**234**) and its corresponding acetate, 12-dehydroxy-16-deacetoxy-22-acetoxy-scalarafuran (**235**), were identified in the sponge *Smenospongia* sp. from Soheuksan Island (Korea). These compounds display antimicrobial activity and strong cytotoxicity against the human chronic myelogenous leukemia K562 cell line [209]. Additionally, coscinafuran (**236**) was detected in the MeOH fraction of the sponge *Coscinoderma mathewsi* [210].

**Figure 29:**
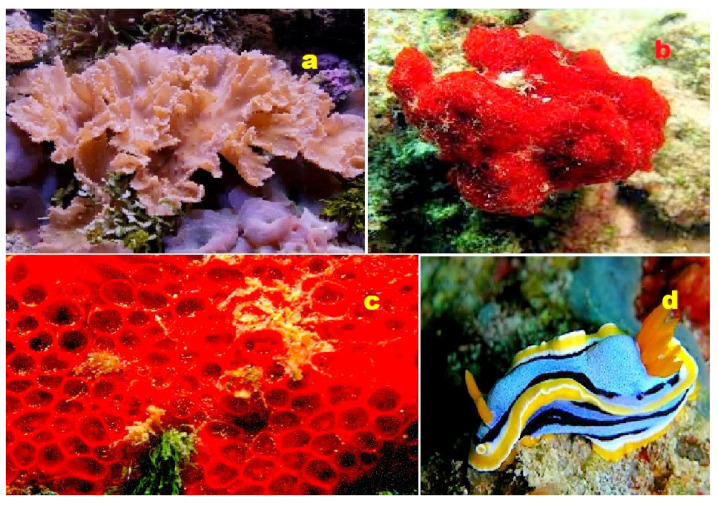
(**a**) The reef soft coral *Sinularia brassica* is a producer of the cytotoxic steroid sinubrasone B (**224**). (**b**) The Caribbean coral reef sponge *Phorbas amaranthus* contains the dimeric steroid amaroxocane B (**227**). (**c**) The Mediterranean sponge *Hamigera hamigera* produces the same sterol dimer, hamigerol B (**227**). (**d**) The nudibranch *Chromodoris sedna* contains two sesterterpenoids, namely sednolide (**237**) and sednolide 22-acetate (**238**). These marine organisms contribute to the production of bioactive compounds with diverse pharmacological properties.

Sednolide (**237**) and sednolide 22-acetate (**238**) were identified in extracts of the nudibranch *Chromodoris sedna*, collected in Baja California (Mexico). A picture of this mollusc is displayed in Figure 29. Sednolide (**237**) demonstrated growth inhibition of the marine bacterium *Vibrio anguillarum* at a concentration of 100 μg/disk [211].

**Figure 30:**
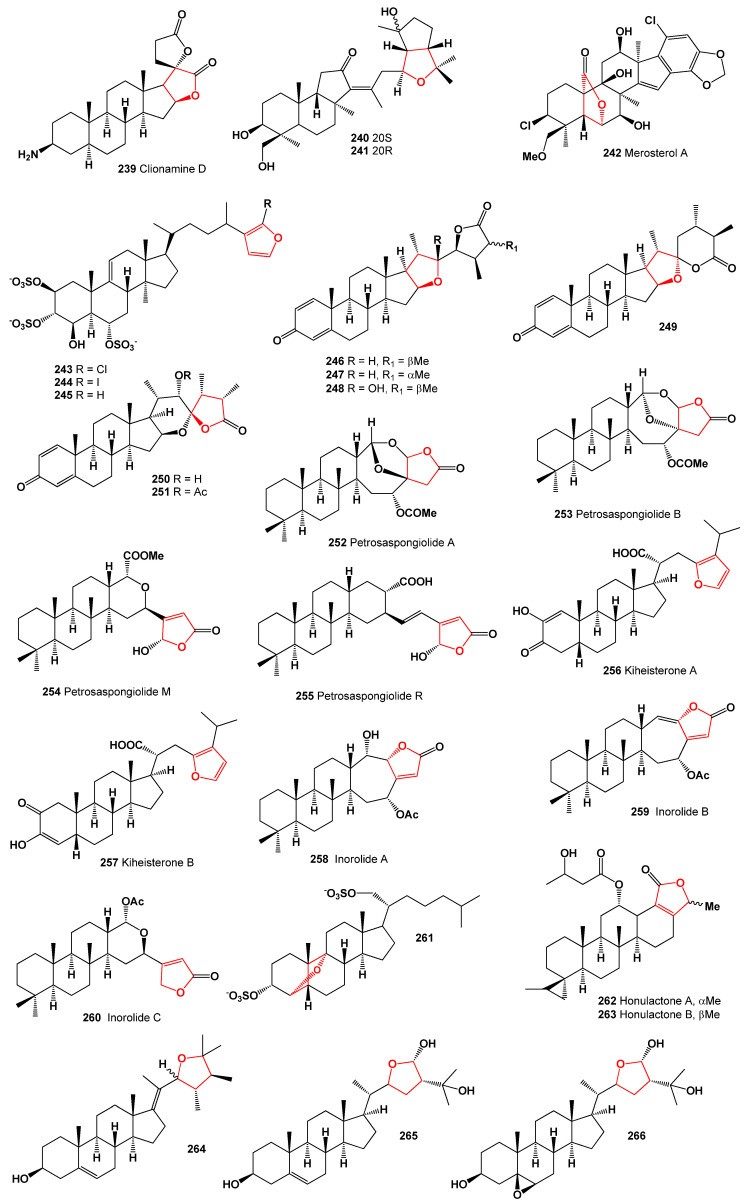
Furanosteroids and triterpenoids derived from marine sources.

A rare and unusual aminosteroid called clionamine D (**239**), with its structure shown in Figure 30 and biological activity presented in Table 10, has been isolated from South African specimens of the sponge *Cliona celata*. This aminosteroid possesses a unique spiro bis-lactone side chain and exhibits cytotoxicity. It also modulates autophagy [212].

Isomalabaricane-type triterpenoids, namely globostelletins P (**240**) and Q (**241**), have been isolated from the marine sponge *Rhabdastrella globostellata*. These triterpenoids, along with the CH_2_Cl_2_ fraction of the sponge, demonstrate inhibitory activities against various human tumor cell lines, including A549 (human lung adenocarcinoma), BGC-823 (human gastric carcinoma), HCT-8 (colonic carcinoma), Bel-7402 (human liver carcinoma), and A2780 (human ovarian carcinoma) [213].

The cyanobacterium *Scytonema* sp. from Bermuda serves as the source of merosterol A (**242**) [177]. Halogenated polar steroids, including chlorine-containing steroid sulfate (**243**), iodinated steroid **244**, and topsentiasterol sulfate D (**245**), have been isolated from the marine sponge *Topsentia* sp. [214,215]. Steroid (**243**) has been shown to effectively inhibit endo-1,3-β-D-glucanase from the marine mollusc *Spisula sachalinensis* [214].

**Figure 31:**
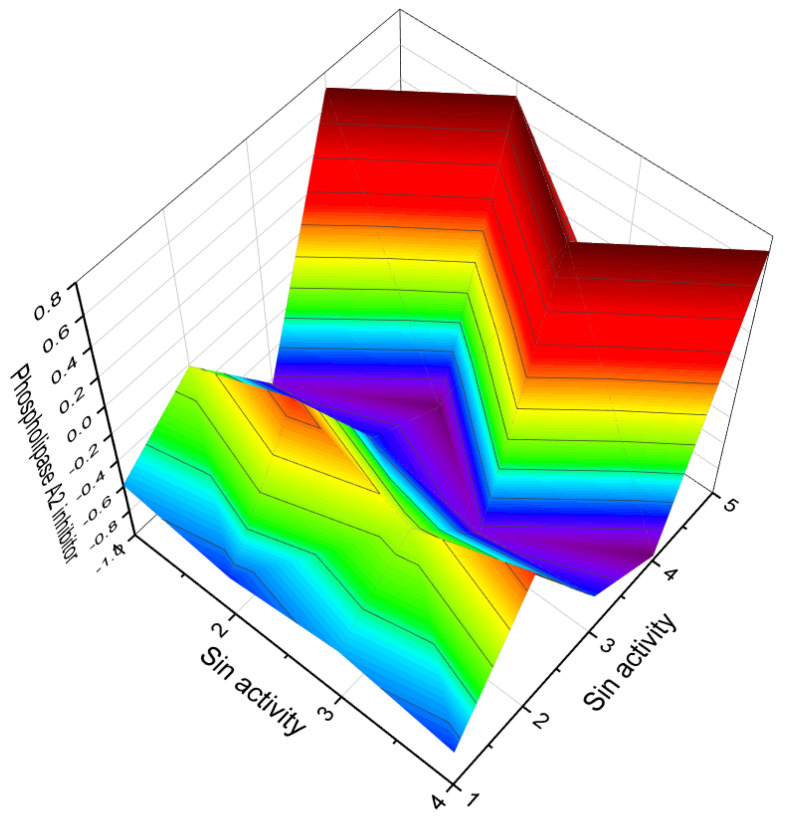
Three-dimensional graph illustrating the predicted and calculated activity of steroids (**254** and **255**) with a confidence level exceeding 90%. These steroids are recognized as phospholipase A2 inhibitors, showcasing their potential pharmacological activity.

A series of steroids known as sinubrasolides A–F (**246**–**251**), which belong to the class of withanolide-type steroids, were isolated from cultured specimens of *Sinularia brassica* from Taiwan [216]. Petrosaspongiolides A (**252**) and B (**253**) were the first cheilantane sesterterpene lactones to be isolated from a New Caledonian sponge initially assigned to the genus *Dactylospongia* but later reclassified as a new genus and species called *Petrosaspongia nigra* [217,218]. Additionally, other petrosasponiolides, namely M (**254**) and R (**255**), isolated from the New Caledonian marine sponge *Petrosaspongia nigra*, exhibited a γ-hydroxybutenolide moiety and a hemiacetal function [219,220]. The 3D graph illustrating the activity of compound **255** is shown in Figure 31.

Cytotoxic steroids named kiheisterones A (**256**) and B (**257**) have been isolated from a sponge of the order *Poecilosclerida* collected along the coast of the island of Maui (Hawaii). These sterols possess an α,β-disubstituted furan in the sidechain, a cis-fused A/B ring, a monoenolized α-di-ketone in the A ring, and a C-21 carboxyl group. Both steroids exhibit mild cytotoxicity against several human tumor cell lines, including A-549 lung carcinoma, HT-29 colon adenocarcinoma, and the P-388 murine lymphocytic leukemia cell line [220]. Furthermore, cytotoxic sesterterpenoids named inorolide A (**258**), B (**259**), and C (**260**) have been isolated from the Japanese nudibranch *Chromodoris inornata* (Chromodorididae) [221].

A unique 5β-steroid disulfate **261** with a distinctive 4α,9α-ether bridge has been isolated from the ophiuroid species *Ophiomastix annulosa* [222]. Furthermore, the marine sponge *Strepsichordaia aliena* from Indonesia has been found to contain the 20,24-bishomoscalarane sesterterpenes honulactones A (**262**) and B (**263**). Both sesterterpenes exhibit cytotoxicity against P-388, A-549, HT-29, and MEL-28 cancer cell lines, with an IC_50_ value of 0.1 μM [223].

**Figure 32:**
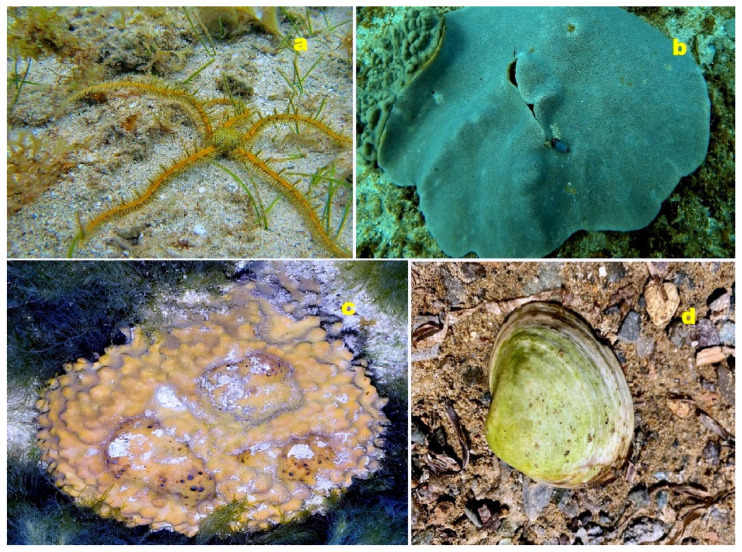
(**a**) The ophiuroid species *Ophiomastix annulosa* serves as a holder of the steroid (**261**). (**b**) The soft coral *Lobophytum depressum* is a producer of steroids (**265**) and (**266**). (**c**) The marine sponge *Rhabdastrella globostellata* contains triterpenoids (**240** and **241**). (**d**) The marine mollusc *Spisula sachalinensis* contains the steroid (**243**). *S. sachalinensis* is a bivalve mollusc found in the Sea of Japan and the Sea of Okhotsk, inhabiting medium- to fine-grained sands at depths ranging from 0.5 to 10 m. It is the largest of the molluscs, reaching a length of approximately 130 mm and weighing around 500 g. This mollusc is considered a delicacy and is actively fished in Japan and Primorsky Krai (Russia) [228].

A sarcosterol derivative, 22,25-epoxy-23,24-dimethylcholesta-5,17(20)-dien-3-ol (**264**), has been discovered in the soft coral *Sinularia mayi* [224]. Additionally, the soft coral Lobophytum depressum contains two steroids: lobophytosterol (**265**), which is a 22,28-epoxyergost-5-ene-3,25,28-triol, and 5,6-epoxy-lobophytosterol (**266**). A picture of this soft coral is displayed in Figure 32 [225,226]. Both steroids possess a double bond at positions 5,6. Lobophytosterol from the soft coral *Lobophytum laevigatum* has demonstrated cytotoxicity against A549 and HL-60 cell lines, with IC_50_ values of 4.5 and 5.6 μM, respectively [227].

## 5. Conclusions

This comprehensive review examines the occurrence of furanosteroids and related isoprenoid lipids in various sources, including fungi, plants, and marine organisms. It provides an in-depth analysis of their biological activity, shedding light on their potential applications and significance in drug discovery. Furanosteroids and related isoprenoid lipids have garnered significant attention due to their diverse biological properties. This review aims to explore the occurrence of these compounds in fungi, plants, and marine organisms, as well as delve into their associated biological activity. By analyzing their medicinal and pharmacological potential, this review highlights the importance of furanosteroids as promising natural products for future research and development. The biological activity of furanosteroids and related isoprenoid lipids constitutes a crucial aspect of their investigation. This review focuses on the diverse range of activities exhibited by these compounds, including but not limited to anti-inflammatory, anticancer, and antimicrobial properties.

## Figures and Tables

**Table 1:** Predicted biological activities of steroids derived from fungi (**2**–**25**).

No.	Dominated Biological Activity (Pa) *	Additional Predicted Activities (Pa) *
**2**	Antifungal (0.912) Anti-inflammatory (0.912) Antiprotozoal (Plasmodium) (0.794) Antibacterial (0.783)	Antineoplastic (0.909) Apoptosis agonist (0.828) Prostate disorders treatment (0.600) Proliferative diseases treatment (0.578)
**3**	Antineoplastic (0.802) Apoptosis agonist (0.647) Chemopreventive (0.573)	Anti-inflammatory (0.629) Antibacterial (0.547) Cytochrome P450 inhibitor (0.503)
**4**	Antineoplastic (0.779) Apoptosis agonist (0.766)	Antibacterial (0.718) Antifungal (0.709)
**5**	Antineoplastic (0.837) Apoptosis agonist (0.704)	Antibacterial (0.745)
**6**	Anti-infertility, female (0.789) Lipid metabolism regulator (0.789)	Antineoplastic (0.756) Apoptosis agonist (0.749)
**7**	Antineoplastic (0.913) Apoptosis agonist (0.907) Inositol-3-kinase inhibitor (0.898)	Lipid metabolism regulator (0.700) Hypolipemic (0.693) Antibacterial (0.642)
**8**	Inositol-4-kinase inhibitor (0.945) Apoptosis agonist (0.865) Inositol-3-kinase inhibitor (0.848)	Antifungal (0.876) Antibacterial (0.733)
**9**	Antineoplastic (0.934) Apoptosis agonist (0.865)	Antifungal (0.881) Antibacterial (0.766)
**10**	Inositol-4-kinase inhibitor (0.949) Inositol-3-kinase inhibitor (0.843) Monoamine oxidase inhibitor (0.837)	Apoptosis agonist (0.899) Antineoplastic (0.879)
**11**	Antineoplastic (0.924) Apoptosis agonist (0.833)	Antifungal (0.876) Antibacterial (0.733)
**12**	Antineoplastic (0.903) Apoptosis agonist (0.807) Prostate disorders treatment (0.800)	Lipid metabolism regulator (0.700) Hypolipemic (0.693)
**13**	Antineoplastic (0.934) Apoptosis agonist (0.907) Prostate disorders treatment (0.866)	Lipid metabolism regulator (0.897) Antifungal (0.881) Hypolipemic (0.793)
**14**	Antiproliferative (0.938) Antineoplastic (0.902) Apoptosis agonist (0.889)	Prostate disorders treatment (0.900) Proliferative diseases treatment (0.878)
**15**	Antiproliferative (0.929) Antineoplastic (0.922)	Prostate disorders treatment (0.876) Proliferative diseases treatment (0.844)
**16**	Antiproliferative (0.915) Apoptosis agonist (0.882)	Prostate disorders treatment (0.854) Proliferative diseases treatment (0.823)
**17**	Antineoplastic (0.877) Apoptosis agonist (0.856)	Ovulation inhibitor (0.698) Anti-inflammatory (0.682)
**18**	Antineoplastic (0.879) Apoptosis agonist (0.876)	Ovulation inhibitor (0.728) Anti-inflammatory (0.717)
**19**	Antineoplastic (0.865) Antiprotozoal (Plasmodium) (0.621)	Antifungal (0.779) Antibacterial (0.747)
**20**	Antineoplastic (0.888)	Antifungal (0.843)
**21**	Antineoplastic (0.910)	Apoptosis agonist (0.877)
**22**	Nitric oxide inhibitor (0.921)	Apoptosis agonist (0.879)
**23**	Nitric oxide inhibitor (0.914) Antifungal (0.862)	Apoptosis agonist (0.867) Antineoplastic (0.844)
**24**	Antineoplastic (0.937)	Apoptosis agonist (0.892)
**25**	Antineoplastic (0.929)	Apoptosis agonist (0.887)

* Only activities with Pa > 0.5 are shown.

**Table 2:** Predicted biological activities of steroids derived from fungi (**26**–**47**).

No.	Dominated Biological Activity (Pa) *	Additional Predicted Activities (Pa) *
**26**	Antineoplastic (0.922) Apoptosis agonist (0.844)	Antifungal (0.887) Antibacterial (0.754)
**27**	Antineoplastic (0.929) Apoptosis agonist (0.839)	Antifungal (0.832) Antibacterial (0.782)
**28**	Antineoplastic (0.921) Apoptosis agonist (0.841)	Antifungal (0.833) Antibacterial (0.755)
**29**	Antineoplastic (0.889)	Antibacterial (0.766)
**30**	Antineoplastic (0.833)	Antibacterial (0.733)
**31**	Hepatoprotectant (0.860) Kidney function stimulant (0.719)	Muscle relaxant (0.599) Spasmolytic (0.587)
**32**	Hepatoprotectant (0.875) Kidney function stimulant (0.686)	Muscle relaxant (0.612) Spasmolytic (0.595)
**33**	Hepatoprotectant (0.839) Kidney function stimulant (0.719)	Muscle relaxant (0.782)
**34**	Antiprotozoal (Plasmodium) (0.718)	Anti-inflammatory (0.778)
**35**	Antiprotozoal (0.657)	Anti-inflammatory (0.766)
**36**	Antiprotozoal (Plasmodium) (0.947) Antiprotozoal (Leishmania) (0.888) Antiprotozoal (0.879)	Antineoplastic (0.866) Apoptosis agonist (0.795) Chemopreventive (0.722)
**37**	Antiprotozoal (Plasmodium) (0.949) Antiprotozoal (Leishmania) (0.868) Antiprotozoal (0.823)	Antineoplastic (0.859) Apoptosis agonist (0.791) Chemopreventive (0.717)
**38**	Antiprotozoal (Plasmodium) (0.927) Antiprotozoal (Leishmania) (0.914) Antiprotozoal (0.884)	Antineoplastic (0.843) Apoptosis agonist (0.747) Chemopreventive (0.698)
**39**	Antiprotozoal (Plasmodium) (0.912) Antiprotozoal (Leishmania) (0.886) Antiprotozoal (0.778)	Antineoplastic (0.876) Chemopreventive (0.773)
**40**	Antiprotozoal (Plasmodium) (0.955) Antiprotozoal (Leishmania) (0.948) Antiprotozoal (0.903)	Chemopreventive (0.903) Antineoplastic (0.822) Apoptosis agonist (0.747)
**41**	Antiprotozoal (0.822)	Anti-inflammatory (0.712)
**42**	Antibacterial (0.883) Antifungal (0.876)	Laxative (0.735) Anti-eczematic (0.717)
**43**	Antibacterial (0.907) Antifungal (0.865)	Laxative (0.722) Anti-eczematic (0.686)
**44**	Antibacterial (0.885) Antifungal (0.876)	Anti-inflammatory (0.712)
**45**	Antibacterial (0.912) Antifungal (0.875)	Anti-eczematic (0.638) Anti-inflammatory (0.612)
**46**	Antibacterial (0.901) Antifungal (0.867)	Anti-eczematic (0.689)
**47**	Antibacterial (0.889) Antifungal (0.882)	Anti-eczematic (0.654)

* Only activities with Pa > 0.5 are shown.

**Table 3:** Predicted biological activities of steroids derived from fungi (**48**–**72**).

No.	Dominated Biological Activity (Pa) *	Additional Predicted Activities (Pa) *
**48**	Antibacterial (0.856) Antifungal (0.811)	Antiviral (0.578)
**49**	Nitric oxide inhibitor (0.906) Apoptosis agonist (0.667)	Antineoplastic (0.764)
**50**	Nitric oxide inhibitor (0.874) Apoptosis agonist (0.685)	Antineoplastic (0.785)
**51**	Plant growth inhibitor (0.876)	Antifungal (0.732)
**52**	Plant growth inhibitor (0.855)	Antifungal (0.689)
**53**	Antibacterial (0.769) Antifungal (0.716)	Antiviral (0.577)
**54**	Nitric oxide inhibitor (0.779)	Antibacterial (0.686)
**55**	Nitric oxide inhibitor (0.812)	Antibacterial (0.677)
**56**	Antineoplastic (0.755)	Anti-inflammatory (0.612)
**57**	Antineoplastic (0.734)	Antifungal (0.521)
**58**	Antibacterial (0.772) Antifungal (0.631)	Antiviral (0.589)
**59**	Antibacterial (0.856)	Antiviral (0.618)
**60**	Antifungal (0.789) Antibacterial (0.634)	Antiviral (0.611)
**61**	Antineoplastic (0.785)	Anti-inflammatory (0.622)
**62**	Cytoprotectant (0.758)	
**63**	Antibacterial (0.882)	Antifungal (0.668)
**64**	Antibacterial (0.872)	Antifungal (0.675)
**65**	Antibacterial (0.868)	Antifungal (0.712)
**66**	Antibacterial (0.856)	Antifungal (0.682)
**67**	Antimicrobial (0.902) Antibacterial (0.856)	Antifungal (0.754)
**68**	Antibacterial (0.911)	Antimicrobial (0.773)
**69**	Antiprotozoal (0.886) Antibacterial (0.847)	Antimicrobial (0.638)
**70**	Antineoplastic (0.772)	Antifungal (0.722)
**71**	Antineoplastic (0.882) Chemopreventive (0.791)	Anti-inflammatory (0.634)
**72**	Antineoplastic (0.913) Chemopreventive (0.882) Apoptosis agonist (0.798)	Antiviral (0.832) Anti-inflammatory (0.711)

* Only activities with Pa > 0.5 are shown.

**Table 4:** Predicted biological activities of steroids derived from fungi (**73**–**101**).

No.	Dominated Biological Activity (Pa) *	Additional Predicted Activities (Pa) *
**73**	Antineoplastic (0.884)	Antifungal (0.621)
**74**	Antineoplastic (0.975) Apoptosis agonist (0.889) Chemopreventive (0.887)	Lipid metabolism regulator (0.858) Hypolipemic (0.677)
**75**	Antineoplastic (0.968) Chemopreventive (0.882) Apoptosis agonist (0.798)	Lipid metabolism regulator (0.897) Hypolipemic (0.693)
**76**	Antineoplastic (0.973) Apoptosis agonist (0.798) Chemopreventive (0.882)	Lipid metabolism regulator (0.872) Hypolipemic (0.654)
**77**	Antineoplastic (0.865)	Antifungal (0.633)
**78**	Antineoplastic (0.834)	Antifungal (0.611)
**79**	Antineoplastic (0.844)	Anti-inflammatory (0.666)
**80**	Antineoplastic (0.851)	Antifungal (0.597)
**81**	Antiproliferative (0.834)	Cytotoxic (0.658)
**82**	Antiproliferative (0.812)	Anti-inflammatory (0.645)
**83**	Antiproliferative (0.876) Antineoplastic (0.875)	Cytotoxic (0.745) Antifungal (0.677)
**84**	Antiproliferative (0.876)	Anti-inflammatory (0.668)
**85**	Antiproliferative (0.924) Cytotoxic (0.892)	Cytotoxic (0.821) Antifungal (0.734)
**86**	Antiproliferative (0.902) Cytotoxic (0.883)	Cytotoxic (0.833) Antifungal (0.680)
**87**	Antineoplastic (0.823)	Anti-inflammatory (0.619)
**88**	Antiproliferative (0.831)	Anti-inflammatory (0.644)
**89**	Antiproliferative (0.849)	Hypolipemic (0.623)
**90**	Antineoplastic (0.736)	Antifungal (0.567)
**91**	Antineoplastic (0.729)	Antifungal (0.564)
**92**	Antineoplastic (0.751)	Antifungal (0.592)
**93**	Antineoplastic (0.722)	Antimicrobial (0.612)
**94**	Antineoplastic (0.733)	Antimicrobial (0.622)
**95**	Antineoplastic (0.728)	Antimicrobial (0.641)
**96**	Antineoplastic (0.802)	Antimicrobial (0.632)
**97**	Allergic conjunctivitis treatment (0.687)	Antifungal (0.566)
**98**	Allergic conjunctivitis treatment (0.705)	Antifungal (0.587)
**99**	Antineoplastic (0.902) Antiproliferative (0.883) Apoptosis agonist (0.870)	Cytotoxic (0.844) Anti-inflammatory (0.634)
**100**	Antibacterial (0.903)	Antimicrobial (0.734)
**101**	Antibacterial (0.868)	Antimicrobial (0.698)

* Only activities with Pa > 0.5 are shown.

**Table 5:** Predicted biological activities of steroids derived from plants (**102**–**127**).

No.	Dominated Biological Activity (Pa) *	Additional Predicted Activities (Pa) *
**102**	Antineoplastic (0.879) Chemopreventive (0.683) Apoptosis agonist (0.641)	Anti-inflammatory (0.840) Antifungal (0.671)
**103**	Antineoplastic (0.851) Chemopreventive (0.662) Apoptosis agonist (0.637)	Anti-inflammatory (0.790) Antifungal (0.653)
**104**	Analgesic (0.761) Antitussive (0.652)	Respiratory analeptic (0.744) Oxygen scavenger (0.651)
**105**	Antiprotozoal (Plasmodium) (0.869) Antiprotozoal (0.832)	Antineoplastic (0.798) Apoptosis agonist (0.795)
**106**	Antiprotozoal (Plasmodium) (0.939) Antiprotozoal (Leishmania) (0.891) Antiprotozoal (0.883)	Antineoplastic (0.874) Apoptosis agonist (0.765) Chemopreventive (0.703)
**107**	Antiprotozoal (Plasmodium) (0.954) Antiprotozoal (Leishmania) (0.912) Antiprotozoal (0.889)	Antineoplastic (0.911) Apoptosis agonist (0.893) Chemopreventive (0.791)
**108**	Antiprotozoal (Plasmodium) (0.941) Antiprotozoal (Leishmania) (0.866) Antiprotozoal (0.836)	Antineoplastic (0.876) Apoptosis agonist (0.795) Chemopreventive (0.722)
**109**	Antiprotozoal (Plasmodium) (0.940) Antiprotozoal (Leishmania) (0.848) Antiprotozoal (0.842)	Antineoplastic (0.868) Apoptosis agonist (0.794) Chemopreventive (0.713)
**110**	Antineoplastic (0.955) Apoptosis agonist (0.802)	Antiviral (0.818) Anti-inflammatory (0.721)
**111**	Antineoplastic (0.946) Apoptosis agonist (0.766)	Antiviral (0.832) Anti-inflammatory (0.734)
**112**	Antineoplastic (0.932) Apoptosis agonist (0.778)	Antiviral (0.822)
**113**	Antineoplastic (0.829) Cytochrome P450 inhibitor (0.691)	Anti-inflammatory (0.790) Antimitotic (0.592)
**114**	Antineoplastic (0.867) Cytochrome P450 inhibitor (0.748)	Anti-inflammatory (0.801) Antimitotic (0.622)
**115**	Antineoplastic (0.793)	Anti-inflammatory (0.715)
**116**	Antiprotozoal (Plasmodium) (0.707) Antiprotozoal (0.697)	Antineoplastic (0.707) Apoptosis agonist (0.570)
**117**	Antineoplastic (0.872) Apoptosis agonist (0.712)	Antiprotozoal (Plasmodium) (0.746) Antiprotozoal (0.703)
**118**	Antineoplastic (0.854) Apoptosis agonist (0.743)	Antiprotozoal (Plasmodium) (0.721) Antiprotozoal (0.693)
**119**	Antineoplastic (0.850) Apoptosis agonist (0.691) Cytostatic (0.683)	Hepatoprotectant (0.767) Immunosuppressant (0.712) Anti-hypercholesterolemic (0.566)
**120**	Antineoplastic (0.855) Apoptosis agonist (0.697)	Hepatoprotectant (0.768) Immunosuppressant (0.718)
**121**	Antineoplastic (0.858) Apoptosis agonist (0.690)	Hepatoprotectant (0.753) Anti-hypercholesterolemic (0.584)
**122**	Antineoplastic (0.860) Apoptosis agonist (0.699)	Hepatoprotectant (0.755) Anti-hypercholesterolemic (0.616)
**123**	Antiviral (0.832)	Anti-inflammatory (0.721)
**124**	Antiviral (0.856)	Anti-inflammatory (0.734)
**125**	Antiviral (0.887) Antiviral (Arbovirus) (0.790)	Antiprotozoal (0.693)
**126**	Antiviral (0.877)	Anti-inflammatory (0.715)
**127**	Antiviral (0.839)	Anti-inflammatory (0.698)

* Only activities with Pa > 0.5 are shown.

**Table 6:** Biological activities of steroids derived from plants (**128**–**154**).

No.	Dominated Biological Activity (Pa) *	Additional Predicted Activities (Pa) *
**128**	Hepatoprotectant (0.784)	Immunosuppressant (0.734)
**129**	Hepatoprotectant (0.772)	Immunosuppressant (0.728)
**130**	Hepatoprotectant (0.777)	Immunosuppressant (0.729)
**131**	Antimicrobial (0.841)	Antibacterial (0.823)
**132**	Antineoplastic (0.823)	Antiprotozoal (0.821)
**133**	Antineoplastic (0.894) Chemopreventive (0.763)	Antiprotozoal (0.865)
**134**	Antineoplastic (0.902) Chemopreventive (0.883)	Antiprotozoal (0.872)
**135**	Antifungal (0.833)	Anti-inflammatory (0.790)
**136**	Antifungal (0.797)	Antimicrobial (0.671)
**137**	Antifungal (0.803)	Antimicrobial (0.666)
**138**	Antimicrobial (0.854)	Antifungal (0.687)
**139**	Antimicrobial (0.798)	Antifungal (0.693)
**140**	Cortisone reductase inhibitor (0.875)	Antineoplastic (0.825)
**141**	Cortisone reductase inhibitor (0.902)	Antineoplastic (0.887)
**142**	Cortisone reductase inhibitor (0.898)	Antineoplastic (0.879)
**143**	Anti-HIV-1 (0.894) Antiviral (0.839)	Anti-inflammatory (0.768)
**144**	Anti-HIV-1 (0.904) Antiviral (0.885)	Anti-inflammatory (0.754)
**145**	Prostate cancer treatment (0.928) Antineoplastic (0.911)	Antimicrobial (0.723)
**146**	Prostate cancer treatment (0.914) Antineoplastic (0.896)	Antimicrobial (0.741)
**147**	Antineoplastic (0.907) Prostate cancer treatment (0.894)	Antimicrobial (0.654)
**148**	Antineoplastic (0.897) Prostate cancer treatment (0.887)	Antimicrobial (0.678)
**149**	Antineoplastic (0.838)	Antimicrobial (0.611)
**150**	Antineoplastic (0.864) Apoptosis agonist (0.723)	Antileukemic (0.822)
**151**	Antineoplastic (0.843) Apoptosis agonist (0.711)	Antifungal (0.733)
**152**	Antineoplastic (0.929) Antimetastatic (0.834)	Antileukemic (0.814)
**153**	Antineoplastic (0.924) Antimetastatic (0.876)	Antileukemic (0.754) Antimicrobial (0.629)
**154**	Antineoplastic (0.932) Antimetastatic (0.839)	Antileukemic (0.724) Antimicrobial (0.623)

* Only activities with Pa > 0.5 are shown.

**Table 7:** Predicted biological activities of steroids derived from plants (**155**–**186**).

No.	Dominated Biological Activity (Pa) *	Additional Predicted Activities (Pa) *
**155**	Antifungal (0.778)	Anti-inflammatory (0.709)
**156**	Antineoplastic (0.918) Prostate cancer treatment (0.915) Antimetastatic (0.734)	Antifungal (0.734) Antimicrobial (0.652)
**157**	Antineoplastic (0.923) Prostate cancer treatment (0.922) Antimetastatic (0.834)	Antifungal (0.736) Antimicrobial (0.655)
**158**	Antineoplastic (0.888) Apoptosis agonist (0.793)	Antifungal (0.713) Antimicrobial (0.654)
**159**	Antineoplastic (0.854) Apoptosis agonist (0.721)	Antifungal (0.743) Antimicrobial (0.664)
**160**	Anti-tuberculosis treatment (0.896)	Antibacterial (0.809)
**161**	Anti-tuberculosis treatment (0.903) Antimicrobial (0.745)	Antibacterial (0.811)
**162**	Anti-tuberculosis treatment (0.937) Antimicrobial (0.772)	Antibacterial (0.854)
**163**	Anti-tuberculosis treatment (0.875)	Antibacterial (0.754)
**164**	Anti-tuberculosis treatment (0.862)	Antibacterial (0.761)
**165**	Acetyl-cholinesterase inhibitor (0.931)	Antimetastatic (0.829)
**166**	Acetyl-cholinesterase inhibitor (0.942)	Antimetastatic (0.842)
**167**	DNA topoisomerase inhibitor (0.928) Antineoplastic (0.916) Antimetastatic (0.863)	Apoptosis agonist (0.834)
**168**	DNA topoisomerase inhibitor (0.917) Antineoplastic (0.922) Antimetastatic (0.821)	Apoptosis agonist (0.871)
**169**	Anti-inflammatory (0.909) Antiviral (0.844)	Antimicrobial (0.688)
**170**	Anti-inflammatory (0.902) Antiviral (0.829)	Antimicrobial (0.679)
**171**	Antineoplastic (0.886)	Apoptosis agonist (0.756)
**172**	Antineoplastic (0.874)	Apoptosis agonist (0.771)
**173**	Antineoplastic (0.859)	Apoptosis agonist (0.754)
**174**	Antineoplastic (0.851)	Apoptosis agonist (0.764)
**175**	Antiproliferative (0.889)	Anti-inflammatory (0.654)
**176**	Antiproliferative (0.882)	Anti-inflammatory (0.659)
**177**	Antiproliferative (0.895)	Anti-inflammatory (0.663)
**178**	Anti-hepatitis C virus (0.912) Antiviral (0.898)	Antimicrobial (0.687)
**179**	Antineoplastic (0.873)	Apoptosis agonist (0.775)
**180**	Antineoplastic (0.869)	Apoptosis agonist (0.768)
**181**	Hepatocellular carcinoma inhibitor (0.897)	Hepatoprotectant (0.860)
**182**	Hepatocellular carcinoma inhibitor (0.906)	Hepatoprotectant (0.873)
**183**	Hepatocellular carcinoma inhibitor (0.911)	Hepatoprotectant (0.869)
**184**	Hepatocellular carcinoma inhibitor (0.889)	Hepatoprotectant (0.856)
**185**	Anti-inflammatory (0.832) Antiviral (0.754)	Antibacterial (0.785)
**186**	Anti-inflammatory (0.834) Antiviral (0.765)	Antibacterial (0.744)

* Only activities with Pa > 0.5 are shown.

**Table 8:** Predicted biological activities of steroids derived from marine sources (**187**–**221**).

No.	Dominated Biological Activity (Pa) *	Additional Predicted Activities (Pa) *
**187**	Antineoplastic (0.913) Antimetastatic (0.786)	Apoptosis agonist (0.619)
**188**	Antineoplastic (0.908) Antimetastatic (0.804)	Apoptosis agonist (0.634)
**189**	Antineoplastic (0.902) Antimetastatic (0.798)	Apoptosis agonist (0.624)
**190**	Cytoprotectant (0.768) Antineoplastic (0.723)	Antimicrobial (0.776)
**191**	Cytoprotectant (0.787)	Antimicrobial (0.685)
**192**	Cytoprotectant (0.766)	Antimicrobial (0.692)
**193**	Antineoplastic (0.900) Apoptosis agonist (0.836)	Antimetastatic (0.698)
**194**	Antineoplastic (0.889) Apoptosis agonist (0.821)	Antimetastatic (0.682)
**195**	Antineoplastic (0.872)	Antiprotozoal (0.765)
**196**	Antineoplastic (0.868)	Antiprotozoal (0.756)
**197**	Antiviral (0.776)	Anti-inflammatory (0.682)
**198**	Antiviral (0.749)	Anti-inflammatory (0.678)
**199**	Antineoplastic (0.773)	Apoptosis agonist (0.653)
**200**	Antibacterial (0.747)	Antifungal (0.683)
**201**	Antibacterial (0.736)	Antifungal (0.651)
**202**	Apoptosis agonist (0.833)	Antineoplastic (0.711)
**203**	Apoptosis agonist (0.824)	Antineoplastic (0.683)
**204**	Antineoplastic (0.744)	Apoptosis agonist (0.621)
**205**	Antineoplastic (0.738)	Apoptosis agonist (0.619)
**206**	Antiallergic (0.728)	Anti-asthmatic (0.671)
**207**	Antiallergic (0.734)	Anti-asthmatic (0.698)
**208**	Antiallergic (0.742)	Anti-asthmatic (0.661)
**209**	Antiallergic (0.741)	Anti-asthmatic (0.676)
**210**	Antineoplastic (0.926)	Antimetastatic (0.855)
**211**	Antineoplastic (0.914)	Antimetastatic (0.836)
**212**	Antiproliferative (0.818)	Antineoplastic (0.654)
**213**	Antiproliferative (0.828)	Antineoplastic (0.662)
**214**	Antiproliferative (0.833)	Antineoplastic (0.659)
**215**	Antiproliferative (0.867)	Antineoplastic (0.678)
**216**	Apoptosis agonist (0.818) Antineoplastic (0.762)	Lipid metabolism regulator (0.638) Anti-inflammatory (0.691)
**217**	Antineoplastic (0.917)	Antimetastatic (0.743)
**218**	Antineoplastic (0.919)	Antimetastatic (0.767)
**219**	Antineoplastic (0.928) Apoptosis agonist (0.854)	Antimetastatic (0.711)
**220**	Antineoplastic (0.931) Apoptosis agonist (0.871)	Antimetastatic (0.721)
**221**	Antineoplastic (0.924)	Antimetastatic (0.734)

* Only activities with Pa > 0.5 are shown.

**Table 9:** Predicted biological activities of steroids derived from marine sources (**222**–**238**).

No.	Dominated Biological Activity (Pa) *	Additional Predicted Activities (Pa) *
**222**	Antiviral (0.788) Antiviral (Arbovirus) (0.750)	Anti-inflammatory (0.682)
**223**	Allergic conjunctivitis treatment (0.705)	Antifungal (0.637)
**224**	Antineoplastic (0.898) Apoptosis agonist (0.834)	Antimetastatic (0.723)
**225**	Antineoplastic (0.918) Apoptosis agonist (0.856)	Antimetastatic (0.743)
**226**	Cathepsin B inhibitor (0.889)	Apoptosis agonist (0.698)
**227**	Antifeedant (0.867)	Antifungal (0.703)
**228**	Protein kinase C inhibitor (0.921) Antineoplastic (0.834) Antimetastatic (0.748)	Antiviral (0.829) Autoimmune disorders treatment (0.619)
**229**	Antineoplastic (0.924) Apoptosis agonist (0.745)	Antifungal (0.698)
**230**	Antineoplastic (0.931) Apoptosis agonist (0.805)	Anti-inflammatory (0.654)
**231**	Antineoplastic (0.918) Apoptosis agonist (0.764)	Anti-inflammatory (0.662)
**232**	Antineoplastic (0.932) Chemopreventive (0.738)	Apoptosis agonist (0.768)
**233**	Antineoplastic (0.929) Chemopreventive (0.787)	Apoptosis agonist (0.729)
**234**	Antineoplastic (0.943) Chemopreventive (0.818)	Apoptosis agonist (0.815)
**235**	Antimicrobial (0.886)	Antifungal (0.687)
**236**	Antineoplastic (0.943) Antimicrobial (0.922)	Apoptosis agonist (0.898) Antifungal (0.847)
**237**	Antibacterial (0.921)	Antifungal (0.727)
**238**	Antibacterial (0.765)	

* Only activities with Pa > 0.5 are shown.

**Table 10:** Predicted biological activities of steroids derived from marine sources (**239**–**266**).

No.	Dominated Biological Activity (Pa) *	Additional Predicted Activities (Pa) *
**239**	Antineoplastic (0.913) Apoptosis agonist (0.886)	Antimetastatic (0.829)
**240**	Antineoplastic (0.922) Chemopreventive (0.731) Anticarcinogenic (0.698)	Apoptosis agonist (0.876)
**241**	Antineoplastic (0.929) Chemopreventive (0.720) Anticarcinogenic (0.678)	Apoptosis agonist (0.854)
**242**	Antineoplastic (0.889) Anticarcinogenic (0.718)	Chemopreventive (0.683)
**243**	Antibacterial (0.881)	Antifungal (0.683)
**244**	Antibacterial (0.879)	Antifungal (0.627)
**245**	Antibacterial (0.902)	Antifungal (0.638)
**246**	Antineoplastic (0.938) Apoptosis agonist (0.834)	Chemopreventive (0.712)
**247**	Antineoplastic (0.886)	Apoptosis agonist (0.726)
**248**	Antineoplastic (0.887)	Apoptosis agonist (0.734)
**249**	Antineoplastic (0.889)	Apoptosis agonist (0.722)
**250**	Antineoplastic (0.900)	Apoptosis agonist (0.783)
**251**	Antineoplastic (0.911)	Apoptosis agonist (0.803)
**252**	Antineoplastic (0.935) Apoptosis agonist (0.814) Chemopreventive (0.756)	Antimetastatic (0.820)
**253**	Antineoplastic (0.929) Apoptosis agonist (0.821) Chemopreventive (0.744)	Antimetastatic (0.822)
**254**	Phospholipase A2 inhibitor (0.923) Antibacterial (0.883)	Anti-inflammatory (0.689)
**255**	Phospholipase A2 inhibitor (0.827) Antibacterial (0.757)	Anti-inflammatory (0.683)
**256**	Antineoplastic (0.884) Apoptosis agonist (0.756)	Antimetastatic (0.688)
**257**	Antineoplastic (0.891) Apoptosis agonist (0.785)	Antimetastatic (0.679)
**258**	Antineoplastic (0.814)	Anti-inflammatory (0.661)
**259**	Antineoplastic (0.828)	Anti-inflammatory (0.658)
**260**	Antineoplastic (0.832)	Anti-inflammatory (0.650)
**261**	Antineoplastic (0.915) Antimetastatic (0.839)	Chemopreventive (0.672)
**262**	Antineoplastic (0.912) Antimetastatic (0.821)	Chemopreventive (0.669)
**263**	Antineoplastic (0.910) Apoptosis agonist (0.752)	Antimetastatic (0.712)
**264**	Antineoplastic (0.921) Apoptosis agonist (0.774)	Antimetastatic (0.729)
**265**	Antineoplastic (0.905) Apoptosis agonist (0.739)	Antimetastatic (0.704)
**266**	Antineoplastic (0.918) Apoptosis agonist (0.699)	Antimetastatic (0.687)

* Only activities with Pa > 0.5 are shown.

## Data Availability

Not applicable.
